# A dynamic structural unit of phase-separated heterochromatin protein 1α as revealed by integrative structural analyses

**DOI:** 10.1093/nar/gkaf154

**Published:** 2025-03-24

**Authors:** Ayako Furukawa, Kento Yonezawa, Tatsuki Negami, Yuriko Yoshimura, Aki Hayashi, Jun-ichi Nakayama, Naruhiko Adachi, Toshiya Senda, Kentaro Shimizu, Tohru Terada, Nobutaka Shimizu, Yoshifumi Nishimura

**Affiliations:** Graduate School of Medical Life Science, Yokohama City University, 1-7-29 Suehiro-cho, Tsurumi-ku, Yokohama, Kanagawa 230-0045, Japan; Graduate School of Agriculture, Kyoto University, Kitashirakawa Oiwake-cho, Sakyo-ku, Kyoto 606-8502, Japan; Structural Biology Research Center, Institute of Materials Structure Science, High Energy Accelerator Research Organization (KEK), 1-1 Oho, Tsukuba, Ibaraki 305-0801, Japan; Center for Digital Green-innovation (CDG), Nara Institute of Science and Technology (NAIST), 8916-5 Takayama, Ikoma, Nara 630-0192, Japan; Department of Biotechnology, Graduate School of Agricultural and Life Sciences, the University of Tokyo, 1-1-1 Yayoi, Bunkyo-ku, Tokyo 113-8657, Japan; Division of Chromatin Regulation, National Institute for Basic Biology, Okazaki 444-8585, Japan; Division of Chromatin Regulation, National Institute for Basic Biology, Okazaki 444-8585, Japan; Division of Chromatin Regulation, National Institute for Basic Biology, Okazaki 444-8585, Japan; Basic Biology Program, The Graduate Institute for Advanced Studies, SOKENDAI, Okazaki 444-8585, Japan; Structural Biology Research Center, Institute of Materials Structure Science, High Energy Accelerator Research Organization (KEK), 1-1 Oho, Tsukuba, Ibaraki 305-0801, Japan; Structural Biology Research Center, Institute of Materials Structure Science, High Energy Accelerator Research Organization (KEK), 1-1 Oho, Tsukuba, Ibaraki 305-0801, Japan; Department of Biotechnology, Graduate School of Agricultural and Life Sciences, the University of Tokyo, 1-1-1 Yayoi, Bunkyo-ku, Tokyo 113-8657, Japan; Department of Mathematical and Physical Sciences, Faculty of Science, Japan Women’s University, 2-8-1 Mejirodai, Bunkyo-ku, Tokyo 112-0015, Japan; Department of Biotechnology, Graduate School of Agricultural and Life Sciences, the University of Tokyo, 1-1-1 Yayoi, Bunkyo-ku, Tokyo 113-8657, Japan; Photon Factory, Institute of Materials Structure Science, High Energy Accelerator Research Organization (KEK), 1-1 Oho, Tsukuba, Ibaraki 305-0801, Japan; RIKEN SPring-8 Center, 1-1-1 Kouto, Sayo-cho, Sayo-gun, Hyogo 679-5148, Japan; Graduate School of Medical Life Science, Yokohama City University, 1-7-29 Suehiro-cho, Tsurumi-ku, Yokohama, Kanagawa 230-0045, Japan

## Abstract

The heterochromatin protein HP1α consists of an N-terminal disordered tail (N-tail), chromodomain (CD), hinge region (HR), and C-terminal chromo shadow domain (CSD). While CD binds to the lysine9-trimethylated histone H3 (H3K9me_3_) tail in nucleosomes, CSD forms a dimer bridging two nucleosomes with H3K9me_3_. Phosphorylation of serine residues in the N-tail enhances both H3K9me_3_ binding and liquid–liquid phase separation (LLPS) by HP1α. We have used integrative structural methods, including nuclear magnetic resonance, small-angle X-ray scattering (SAXS), and multi-angle-light scattering combined with size-exclusion chromatography, and coarse-grained molecular dynamics simulation with SAXS, to probe the HP1α dimer and its CSD deletion monomer. We show that dynamic intra- and intermolecular interactions between the N-tails and basic segments in CD and HR depend on N-tail phosphorylation. While the phosphorylated HP1α dimer undergoes LLPS via the formation of aggregated multimers, the N-tail phosphorylated mutant without CSD still undergoes LLPS, but its structural unit is a dynamic intermolecular dimer formed via the phosphorylated N-tail and a basic segment at the CD end. Furthermore, we reveal that mutation of this basic segment in HP1α affects the size of heterochromatin foci in cultured mammalian cells, suggesting that this interaction plays an important role in heterochromatin formation *in vivo*.

## Graphical Abstract

**Graphical Abstract:**
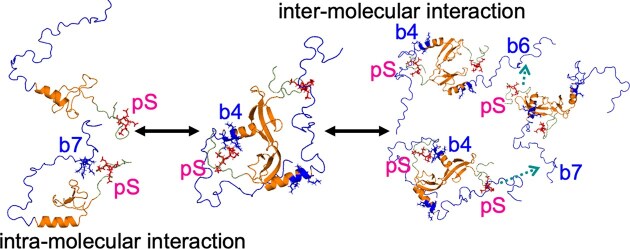

## Introduction

Folding of chromatin into a condensed higher order structure, so-called heterochromatin, is critical for genomic stability and transcriptional silencing [1]. Methylation of the lysine 9 residue on histone H3 (H3K9me) is known as an indispensable hallmark of heterochromatin formation [2, 3]. Heterochromatin protein 1 (HP1) specifically binds to chromatin containing tri-methylated histone H3 (H3K9me_3_) [4–6]. Three HP1 isoforms, HP1α, HP1β, and HP1γ, have been identified in mammalian cells [7]. These HP1 isoforms contain an N-terminal chromodomain (CD), which binds to H3K9me_3_ [8–10], and a C-terminal chromo shadow domain (CSD), which is used for HP1 dimerization. CSD also provides an interface for recruiting diverse target proteins [11]. The HP1 dimer can bridge two nucleosomes via H3K9me_3_, stabilizing the higher order chromatin structure [12–14].

In HP1, the N-terminal tail (N-tail) before CD and the hinge region (HR) connecting CD and CSD are intrinsically disordered regions (IDRs). Among the three HP1 isoforms, CD and CSD are well conserved, but there is less conservation of the disordered N-tail and HR [15]. In particular, HP1α has four successive serine residues in the N-tail that are constitutively phosphorylated by casein kinase 2 (CK2) *in vivo* [16]. The phosphorylated N-tail strongly enhances binding affinity between HP1α CD and H3K9me_3_, and increases specificity for the H3K9me_3-_marked nucleosome [16–18].

In our previous study, we integrated nuclear magnetic resonance (NMR), small-angle-X-ray-scattering (SAXS), and molecular dynamics (MD) to probe HP1α fragments comprising the N-tail and CD, which showed that the unphosphorylated N-tail dynamically fluctuates to interfere with binding between H3K9me_3_ and CD, while the phosphorylated N-tail adopts a rather extended structure, allowing H3K9me_3_ to bind to CD and enhancing the binding by electrostatic interactions with the basic segment of the H3 N-tail that follows the H3K9me_3_ region [17]. However, the tertiary structure of full-length HP1α with or without N-terminal phosphorylation has remained elusive because HP1α forms a dimer via two monomer CSDs, each of which contains two flexible IDRs, resulting in dynamic and complicated interactions. Recently, the cryo-electron microscopy structure of the H3K9me_3_-containing di-nucleosome complexed with unphosphorylated HP1α has been reported; however, the N-tail, CD, and HR are not observed in this structure [19].

In addition, it has been reported that the phosphorylated N-tail promotes liquid–liquid phase separation (LLPS) by HP1α *in vitro* [20]. HP1 proteins from different species have also been reported to undergo LLPS *in vitro* [21, 22]. Although HP1α interacts with nucleosomes, ligand proteins, and DNA/RNA, showing complicated relations between its ability to undergo LLPS and formation of the heterochromatic structure *in vivo* [21–27], the importance of LLPS in chromatin function has been proposed [20–32]; as a result, mechanistic studies of LLPS in the nucleus have attracted much attention from researchers [33–37].

**Figure 1.**
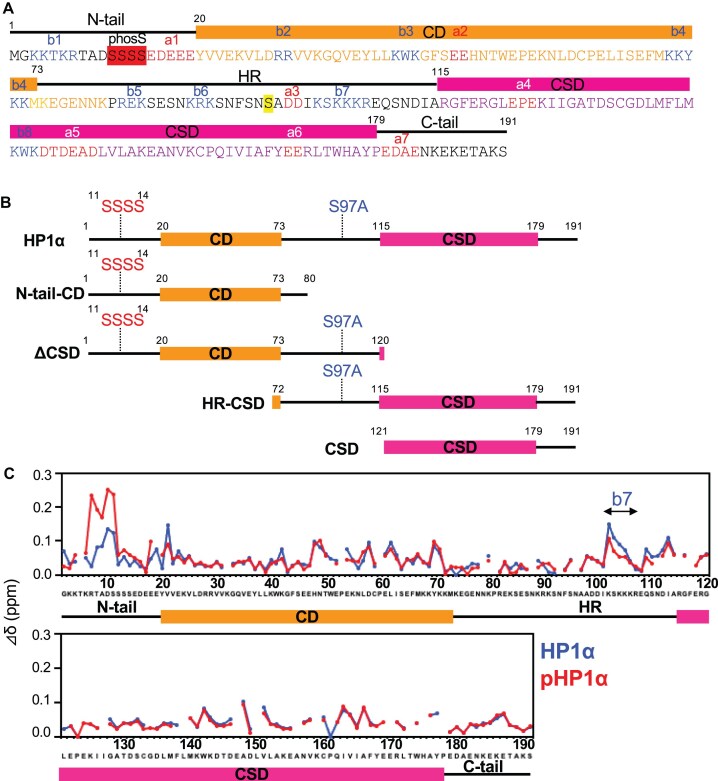
Comparison of phosphorylated and unphosphorylated HP1α by NMR. (**A**) Sequence of HP1α and numbering of basic and acidic segments. Phosphorylation sites are shown as phosS. (**B**) Schematic representations of the HP1α mutants used for NMR, SAXS, and MD experiments. Mutants phosphorylated at the serine residues marked as SSSS are defined as pHP1α, pN-tail-CD, or pΔCSD. The mutated amino acid residue is shown as S97A. (**C**) Chemical shift differences (Δδ) between 500 and 50 mM NaCl.

LLPS is brought about by dynamic multivalent interactions, such as hydrophobic, electrostatic, cation–pi, and pi–pi contacts [38, 39]. A study based on cross-linking and mass spectrometry revealed multiple inter- and intra-subunit within an HP1α dimer [20]. Because HP1α contains acidic and basic segments (Fig. 1A) with two IDRs and forms a dimer, it is likely to adopt complex and dynamic intra- and intermolecular electrostatic interactions. An earlier study showed that high salt concentration (500 mM NaCl) hampers the ability of HP1α to undergo LLPS, suggesting that electrostatic intermolecular interactions are required for this process [20]. Moreover, high concentrations of HP1α are required for LLPS [20]. A model of LLPS formation has been proposed based on size-exclusion chromatography coupled with SAXS (SEC-SAXS) at a relatively high concentration of phosphorylated HP1α (pHP1α), which suggests that, at 150 μM, the unphosphorylated HP1α dimer does not form oligomers, but the pHP1α dimer forms higher order oligomers mediated via its elongated structure [20]; the two elongated phosphorylated N-tails of the pHP1α dimer seem to interact with the neighboring pHP1α dimers, causing LLPS. However, details of the molecular mechanism of LLPS remain elusive.

To elucidate the molecular mechanism of LLPS by HP1α, here we have utilized SEC-SAXS, multi-angle light scattering combined with size-exclusion chromatography (SEC-MALS), and coarse-grained molecular dynamics (CGMD) simulation with SAXS (CGMD-SAXS) to clarify the relationship between LLPS mediated by phosphorylation and the intra- and intermolecular interactions of HP1α and its CSD deletion mutant (ΔCSD). Collectively, our integrative structural analyses reveal that the dynamic dimer by electrostatic interactions between N-tail and CD or HR is critical for HP1α to undergo LLPS.

## Materials and methods

### Plasmid construction

DNA fragments encoding human HP1α (residues 1–80, N-tail-CD; 1–120, ΔCSD; 72–191, HR-CSD; 121–191, CSD) were inserted into the NdeI–BamHI sites of a modified pET15b vector, in which the thrombin cleavage site was replaced by the PreScission Protease cleavage site [19] (Fig. 1B). Plasmids for mutants (S97A and K68A–K72A) were generated by site-directed mutagenesis using a KOD-Plus-Mutagenesis Kit (Toyobo, Osaka, Japan) in accordance with the manufacturer’s protocol. To express EGFP-fused WT and mutant HP1α in NIH3T3 cells, mouse HP1α cDNA was polymerase chain reaction (PCR)-amplified from an NIH3T3 cDNA library and cloned into mammalian expression vector pEGFP-C1 (Clontech) [16]. To introduce either S11-14A (SA) or b4KA (b4), or both (SAb4) mutations, the resultant plasmid (mHP1α–WT–pEGFP-C1) was subjected to site-directed mutagenesis as described previously [40].

To express HP1α/Swi6 chimeric proteins in fission yeast cells from the endogenous *swi6*^+^ locus, the *swi6*^+^ coding sequence with its potential promoter and terminator regions was first cloned into pBluescript; five restriction enzyme sites were introduced by site-directed mutagenesis (BamHI, after the ATG codon; NspV, at the N-CD junction; NruI, at the CD-H junction; PmeI, at the H-CSD junction; and PacI, after the stop codon) [16]; and then an *ura4*^+^ marker gene was introduced via the HindIII site (pAL2-UBP). The PCR-amplified DNA fragment for HP1α ΔCSD was introduced into the pAL2-UBP vector via the BamHI and PmeI sites, and SA, b4, and SAb4 mutations were introduced by site-directed mutagenesis. The resultant plasmids were cleaved with MfeI and introduced into the original *swi6*^+^ locus. To replace the WT *swi6*^+^ allele with an allele expressing HP1α/Swi6 chimeric proteins, strains that had lost the *ura4*^+^ gene through internal homologous recombination were isolated by using counter-selective medium containing 5-fluoroorotic acid (FOA). Strains harboring *Kint2*::*ura4*^+^ were constructed using standard genetic crosses.

### Protein expression and purification

All HP1α His-tag fusion proteins were expressed in *Escherichia coli* strain BL21 (DE3) star (Thermo Fisher Scientific) transformed with the relevant pET15b/Amp expression plasmid with or without pRSFduet/Kan (expressing CK2); cells were grown in LB medium or isotope supplemented M9 media at 37°C [17]. For resonance assignment, 2 g/l of [^13^C] glucose and 1 g/l of [^15^N] ammonium chloride were added to the media. For other NMR experiments, 1 g/l of [^15^N] ammonium chloride was used. For full-length HP1α_S97A, D_2_O media was used. When *A*_600_ reached 0.7, 1 mM isopropyl β-d-1-thiogalactopyranoside was added to induce protein expression. The cells were incubated overnight at 15°C and harvested by centrifugation at 6700 *g* for 20 min at 4°C. The cell pellets were sonicated in 20 mM Tris (pH 7.6), 1 M NaCl, 1 mM dithiothreitol (DTT), and then centrifuged at 47 000 *g* for 20 min at 4°C. The supernatant was loaded onto a Ni Sepharose resin column (GE Healthcare), which was washed with 20 mM Tris–HCl (pH 7.6), 1 M NaCl, 1 M DTT, and 10 mM imidazole, and eluted with an imidazole gradient of 10–400 mM. The His-tag was cleaved by HRV3C protease (Fuji Film) in 20 mM Tris–HCl (pH 7.6), 500 mM NaCl, 1 mM DTT, and 1 mM EDTA at 4°C overnight. After removal of HRV3C protease via a Glutathione Sepharose resin column (Cytiva), protein fractions passing through the column were concentrated using an ultrafiltration cartridge (Millipore) and diluted with 20 mM Tris–HCl (pH 7.6) and 1 mM DTT. The resulting solution was loaded onto a HiTrap Heparin column (Cytiva) equilibrated with 20 mM Tris–HCl (pH 7.6), 50 mM NaCl, 1 M DTT, and eluted with an NaCl gradient of 50–1000 mM. The eluted solutions were concentrated and passed through HiLoad 16/60 Superdex 200pg equilibrated in 20 mM sodium phosphate buffer (pH 7.0), 50 or 500 mM NaCl, and 1 mM DTT. Proteins fractions were concentrated using an ultrafiltration cartridge (Millipore). If necessary, phosphorylation of the four serine residues in each protein sample was confirmed by mass spectrometry using a MALDI-TOF AutoflexTM System (Bruker Daltonics).

### NMR spectroscopy

NMR spectra were acquired on AVANCE 600-MHz and AVANCE III HD 950-MHz spectrometers with a triple-resonance TCI cryogenic probe (Bruker BioSpin) at 298 K. The protein concentrations were 0.1–1 mM in 20 mM sodium phosphate buffer (pH 7.0), 50 or 500 mM NaCl, 1 mM DTT, and 5% D_2_O.

### Backbone assignment

Three-dimensional transverse relaxation optimized spectroscopy (TROSY) spectra of HNCO, HN(CA)CO, HNCA, HN(CO)CA, HNCACB, and HN(CO)CACB were measured for sequential assignments of the backbone ^1^H, ^13^C, and ^15^N chemical shifts of HP1α_S97A, pHP1α_S97A; HP1α_N-tail-CD, pHP1α_N-tail-CD; HP1αΔCSD_S97A, pHP1αΔCSD_S97A; HP1α_HR-CSD, pHP1α_HR-CSD; and HP1α_CSD. NMR data were processed by NMRPipe [41], and signal assignments were performed with Magro [42]. NMR data were analyzed by NMRViewJ (One Moon Scientific, Inc.) and PINT [43]. Chemical shift difference (Δδ) was calculated by the equation Δδ = [(Δδ_H_)^2^ + (Δδ_N_/5)^2^]^1/2^, where Δδ_H_ and Δδ_N_ are chemical shift differences of the amide proton and nitrogen atoms, respectively.

### SEC-MALS and SEC-SAXS

SEC-MALS was performed using a DAWN HELEOS II system (Wyatt Technology) in combination with an Alliance 2695 high-performance liquid chromatography (HPLC) system (Waters). Sample concentrations were calculated by using a 2414 Refractive Index (RI) detector (Waters) connected in series downstream of the MALS instrument. Samples were dissolved in 20 mM sodium phosphate buffer (pH 7.0), 1 mM DTT, 50 or 500 mM NaCl, and injected onto a Superdex 200 Increase 10/300 or 3.2/300 column (Cytiva) equilibrated in the same buffer at a flow rate of 0.5 or 0.05 ml/min, respectively; the pre-injection concentrations and injection volumes of the samples are summarized in Supplementary Fig. S3A. Molar masses were calculated in ASTRA 6.1 (Wyatt Technology) by using Rayleigh ratio and differential RI values.

SEC-SAXS data were collected on BL-10C [44] and BL-15A2 [45] at the Photon Factory, KEK (Tsukuba, Japan). The SEC-SAXS experiments were performed using an HPLC systems, Prominence-i and Nexera-i (SHIMADZU), connected to a Superdex 200 Increase 10/300 or 3.2/300 column (Cytiva) equilibrated with the same buffer as that of SEC-MALS at a flow rate of 0.05 or 0.01 ml/min, respectively. Serial scattering images were taken with 20-s exposure on BL-10C and 3-s exposure on BL-15A2 and recorded by a PILATUS3 2M detector (DECTRIS). The 15 images measured before the sample fraction were averaged to obtain a background profile. Fiber spectrometers, QE65pro (Ocean Insight) on BL-10C and QEpro (Ocean Insight) on BL-15A2, mounted at an angle of 45° to the sample cell, were also utilized to obtain the concentration for each scattering image. All scattering images were azimuthally averaged to convert the one-dimensional scattering intensity data. Background subtraction was performed and the scattering intensities were calibrated to the absolute scale by using water as a standard. These processes were carried out using *SAngler* [46]. Scattering profiles above the top half of the elution peaks were averaged by using *MOLASS* [47]. The radius of gyration (*R*_g_) and forward scattering intensity (*I*(0)) were automatically calculated from the Guinier approximation by *MOLASS* and *AUTORG* of *ATSAS* [48]. The pair-distance distribution function, *P*(*r*), was also calculated using *GNOM* of *ATSAS* [49].

The IDRs in HP1α are likely to cause the random existence of different structures in solution; therefore, the Ensemble Optimization Method (EOM) [50] was used to investigate the size distribution of these structures via ATSAS online (https://www.embl-hamburg.de/biosaxs/atsas-online/) [49]. PDB structures 3FDT [51] and 3Q6S [52] were used as crystal structures for the CD and CSD regions. Regions without crystal structures, such as linkers, were calculated from sequence information under conditions set to native-like structures. Based on the basic specification of the EOM, the initially generated structures were output as 10 000 structures, and a genetic algorithm was used to obtain structural variances optimized for each experimental scattering profile. Supplementary Figs S4F, S9F, and S15E show the results of the EOM calculation. In each graph, the dashed lines represent the *D*_max_ distribution before EOM was applied, calculated from the initial 10 000 structural models generated; the solid lines represent the distribution obtained as a result of optimization. Detailed information on the SEC-SAXS experiments and analyses is summarized in Supplementary Table S1. The structure information has been submitted to the Small-Angle-Scattering Biological Data Bank (SASBDB; https://www.sasbdb.org/aboutSASBDB/) [53] under the IDs SASDU23, SASDU33, SASDU43, SASDU53, SASDU63, SASDU73, and SASDU83.

### CGMD simulations

A structural model of dimeric HP1α was constructed by combining two CD (residues 16−80) models and one CSD dimer (residues 111−180) model produced by AlphaFold2 [54] with models of the other regions (i.e. the N- and C-terminal tails and the linker) produced by Modeller 10 [55]. The structural model of ΔCSD was obtained by extracting residues 1–114 from a subunit of the HP1α model.

The MARTINI 2.2 coarse-grained models [56, 57] of HP1α and ΔCSD were generated from the structural models using martinize.py script [58]. The structures of residues 19−74 of CD and residues 113−173 of the CSD dimer were maintained by elastic networks because the structures of these regions were predicted with high confidence scores (pLDDT > 70) (Supplementary Fig. S16). Elastic bonds were applied to backbone bead pairs at a distance of 5−11 Å within CD and the CSD dimer with force constants of 250 and 150 kJ mol^−1^ nm^−2^, respectively. The cutoff distances and the force constants were determined so that the distributions of the RMSDs from the initial structure calculated for the backbone beads of CD and the CSD dimer in a CGMD simulation were comparable to those of the corresponding atoms in an AAMD simulation (see Supplementary Data). The structures of pHP1α and ΔCSD (pΔCSD) were modeled by replacing the beads of serine residues in the N-tail (residues 11−14) with those of phosphorylated serine (see Supplementary Data). Each dimer structure of HP1α and pHP1α was solvated in a box of ∼165 000 CG water molecules. For both ΔCSD and pΔCSD, two different systems, a single-molecule system and a two-molecule system, were constructed. In the single-molecule system, one protein model was solvated in a box of ∼34 900 CG water molecules. In the two-molecule systems, two protein models were randomly placed in a box of 210 Å × 210 Å × 210 Å at a distance of >60 Å from each other using packmol [59]; the models were then solvated with ∼67 500 CG water molecules. Na^+^ and Cl^−^ beads were added to each system at a concentration of 50 mM. Using a modified version of the MARTINI 2.2 force field, CGMD simulations were performed for the systems containing the HP1α and pHP1α models and for the one-molecule and two-molecule systems of ΔCSD and pΔCSD (see Supplementary Data). Details of the CG mapping and the parametrization of phosphorylated serine are also described in Supplementary Data. Each system was energy-minimized and equilibrated for 200 ps with positional restraints. Five production runs of 5 μs were performed in the constant-*NPT* ensembles with different initial velocities. The temperature was kept at 300 K using the velocity-rescaling thermostat [60]. The pressure was kept at 1.0 bar using the Parrinello–Rahman barostat [61]. Electrostatic interactions were calculated using the reaction-field method [62] with a cutoff of 1.1 nm. Van der Waals interactions were calculated with a modified Lennard–Jones potential, where the potential was shifted to zero at the cut-off distance of 1.1 nm. The linear constraint solver (LINCS) algorithm [63, 64] was used to constrain bond lengths. A time step of 20 fs was used. All simulations were performed in GROMACS 2023.5 [65].

### Ensemble reweighting based on SAXS data

To reproduce the experimental SAXS profiles, the structural ensembles produced by the CGMD simulation were reweighted by using the Bayesian maximum entropy (BME) method [66]. The snapshot structures at every 10 ns of the CG production runs were transformed to all-atom models by using the reverse transformation protocol [67]. Subsequently, the reweighting was performed as follows:

1. SAXS profiles were calculated for each snapshot using FoXS [68].
2.
$N$ was set as the number of snapshots. Weights of the trajectory $w = ( {{w_1},{w_{2,}} \cdots {w_N}} )$ were determined to minimize the following expression:
  \begin{equation*}L\left( w \right) = \frac{{{\chi ^2}\left( w \right)}}{2} - \theta S\left( w \right).\end{equation*}

The first term is cost of fitting of the calculated SAX profile to the experimental one:


\begin{equation*}{\chi ^2}\left( w \right) = \mathop \sum \limits_{i = 1}^M {\left( {\frac{{\mathop \sum \nolimits_{j = 1}^N \left( {{w_j}{I_{j,i}}} \right) - {I_{{\mathrm{exp}},i}}}}{{{\sigma _i}}}} \right)^2},\end{equation*}

where ${I_{j,i}}$, ${I_{{\mathrm{exp}},i}}$, and ${\sigma _i}$ are calculated intensities, experimental intensities, and experimental errors, respectively. Index *i* runs over the *M* measured data points.

The second term, $S( w )$, is relative entropy:


\begin{equation*}S\left( w \right) = - \mathop \sum \limits_{j = 1}^N {w_j} \cdot \log \left( {\frac{{{w_j}}}{{w_j^0}}} \right),\end{equation*}

where $w_j^0$ are initial weight, for which the uniform distribution was used here. The contribution of entropy is controlled by parameter *θ*. Here, the parameter *θ* was set to 20, a value at which the effective fraction ${\varphi _{{\mathrm{eff}}}}\;( { = \exp ( {S( w )} )} )$ of snapshots that contribute to the reweighted ensemble with substantial weights was >0.7 for both HP1α and pHP1α (Supplementary Fig. S17).

1. The fitting parameters (*c*_1_, *c*_2_) of the FoXS calculation (step 1) were averaged with weights $w$.
2. SAXS profiles were recalculated for each snapshot using the weighted averages of the parameters.
3. The structural ensemble was reweighted again by BME (step 2) using the SAXS profiles calculated in step 4.

### Cell culture

NIH3T3 cells (no. RCB0150: RIKEN Bioresource Center) were cultured in Dulbecco’s modified Eagle’s medium (DMEM) (Nacali Tesque) supplemented with 10% fetal calf serum (Invitrogen). Transfection of plasmid DNA into NIH3T3 cells was carried out using Lipofectamine 3000 (Invitrogen). After 48 h, the cells were harvested and used for further experiments.

### Microscopy

Cells grown on coverslip-bottomed culture dishes (MatTek) were washed briefly with phosphate-buffered saline (PBS) and incubated with PBS containing 5 μg/ml Hoechst33342 (Invitrogen) for 5–10 min at room temperature. The cells were washed three times with PBS and cell images were acquired with an BZ-9000 (Keyence). The number and area of EGFP–HP1α foci in the nuclei were measured by Fiji after background subtraction and noise removal using a median filter. The measured cell number was 30 for both WT and mutant EGFP–HP1α. Data were evaluated for statistical significance by Mann–Whitney’s *U* test using the R package (https://www.r-project.org.). Beeswarm plots were made using the R and beeswarm packages.

### Silencing assays

Silencing assays were performed as described previously [69]. In brief, cells carrying a silencing marker (*Kint2::ura4*^+^) were grown in yeast extract with adenine (YEA) medium, collected by centrifugation, and resuspended in water. Serial dilutions (10-fold) were prepared and spotted onto nonselective medium or minimal medium containing FOA plates, which were then incubated at 30°C for 2–5 days.

### RT-qPCR analyses

Total RNA was extracted from cells as described previously [69]. RNA samples from each strain were preincubated with RNase-free DNase I (0.4 U/μg RNA; TaKaRa) to remove any residual genomic DNA. Reverse transcription quantitative PCR (RT-qPCR) was performed using the One Step TB Green PrimeScript PLUS RT-PCR Kit (TaKaRa) and a real-time PCR machine (StepOnePlus, Applied Biosystems). The primers used in RT-qPCR are listed in Supplementary Table S2. Data were analyzed using the ΔΔ*C*_t_ method. The signals were normalized to *act1^+^* and represent the fold increase relative to the WT signal. Error bars show the SEM (*n* = 3). *P*-values were calculated using Welch’s *t*-test; **P < 0.01.

## Results

### Intramolecular interactions of HP1α and pHP1α revealed by NMR

By switching between two different NaCl concentrations (50 and 500 mM), we first investigated the electrostatic interactions present in dimeric full-length HP1α and its phosphorylated form (phosphorylation at Ser11, Ser12, Ser13, and Ser14) at a concentration of 150 μM. To avoid CK2-mediated *in vitro* phosphorylation of Ser97, which is barely detected in cells [16, 18, 69], we used a Ser97Ala variant for the NMR, SEC-SAXS, and SEC-MALS analyses. Full-length HP1α_Ser97Ala and its phosphorylated form (Ser11, Ser12, Ser13, and Ser14) are hereafter referred as HP1α and pHP1α, respectively.

The ^1^H-^15^N HSQC spectra of HP1α and pHP1α at 500 mM NaCl were almost identical except for the phosphorylation site of the N-tail (Supplementary Fig. S1A and C). Relative to the high salt condition, both HP1α and pHP1α showed significant chemical shift changes in many regions at 50 mM NaCl, suggesting that HP1α and pHP1α form dynamic electrostatic intra- and/or intermolecular interactions (Fig. 1C, and Supplementary Fig. S1B and C). Here, to enable us to describe the electrostatic interactions between the basic and acidic segments of HP1α, we numbered the segments from a1 to a7 and from b1 to b7, respectively (Fig. 1A). Almost all chemical shift changes observed between the 50 and 500 mM NaCl forms were similar between HP1α and pHP1α, suggesting that HP1α and pHP1α have similar dynamic intramolecular interactions because HP1α is reported to exist as a stable dimer at this condition. However, marked chemical shift differences were observed for HP1α at b7 (Lys102–Lys105) and for pHP1α at the phosphorylated N-tail (Fig. 1C), suggesting that the basic segment b7 (Lys102–Lys105) in HP1α interacts intra- and/or intermolecularly with other acidic regions, while the phosphorylated N-tail in pHP1α interacts intra- and/or intermolecularly with other basic regions.

In addition, between HP1α and pHP1α at 50 mM NaCl, small but significant shift differences were observed for Tyr20 after a1, Lys42 in b3, His48 after a2, Cys59, and Lys89–Ser92 in b6 (Supplementary Fig. S1C and D). For CD, the residues showing significant shift differences are located near the aromatic cage consisting of Tyr20, Trp41, and Phe44 required for H3K9me_3_ binding, which may correlate with the increased affinity of HP1α for H3K9me_3_ upon phosphorylation. Overall, the observed chemical shift changes suggest that HP1α and pHP1α differ somewhat in their intra- and/or intermolecular interaction modes.

To clarify the different interaction modes between the phosphorylated and unphosphorylated N-tail, we examined the N-tail dynamics of HP1α and pHP1α by heteronuclear Overhauser effect (NOE) experiments. The phosphorylated N-tail showed NOE values of ∼0.4, indicating that it probably behaves as an extended string; however, the unphosphorylated N-tail showed reduced values around Thr8–Asp10 and Ser12–Ser14, suggesting a much more flexible string relative to the phosphorylated N-tail (Supplementary Fig. S2).

### Overall structural differences in HP1α and pHP1α revealed by SEC-MALS/SAXS and CGMD-SAXS

We used SEC-MALS and SEC-SAXS of HP1α and pHP1α to confirm variations in overall structure and characteristics arising from differences in their electrostatic interactions. For consistency with the NMR data, NaCl was maintained at 50 and 500 mM to assess the effects of electrostatic interactions.

SEC-MALS analysis confirmed the dimeric structure of each form with a molecular mass of 45.7 and 48.3 kDa for HP1α (loading concentration, 249 μM) and pHP1α (loading concentration, 307 μM), respectively, at 50 mM NaCl (Supplementary Fig. S3A–C), and 44.5 kDa for both HP1α (loading concentration, 111 μM) and pHP1α (loading concentration of 110 μM), at 500 mM NaCl (Supplementary Fig. S3A, D, and E); these results are essentially the same as those reported previously [20].

**Figure 2.**
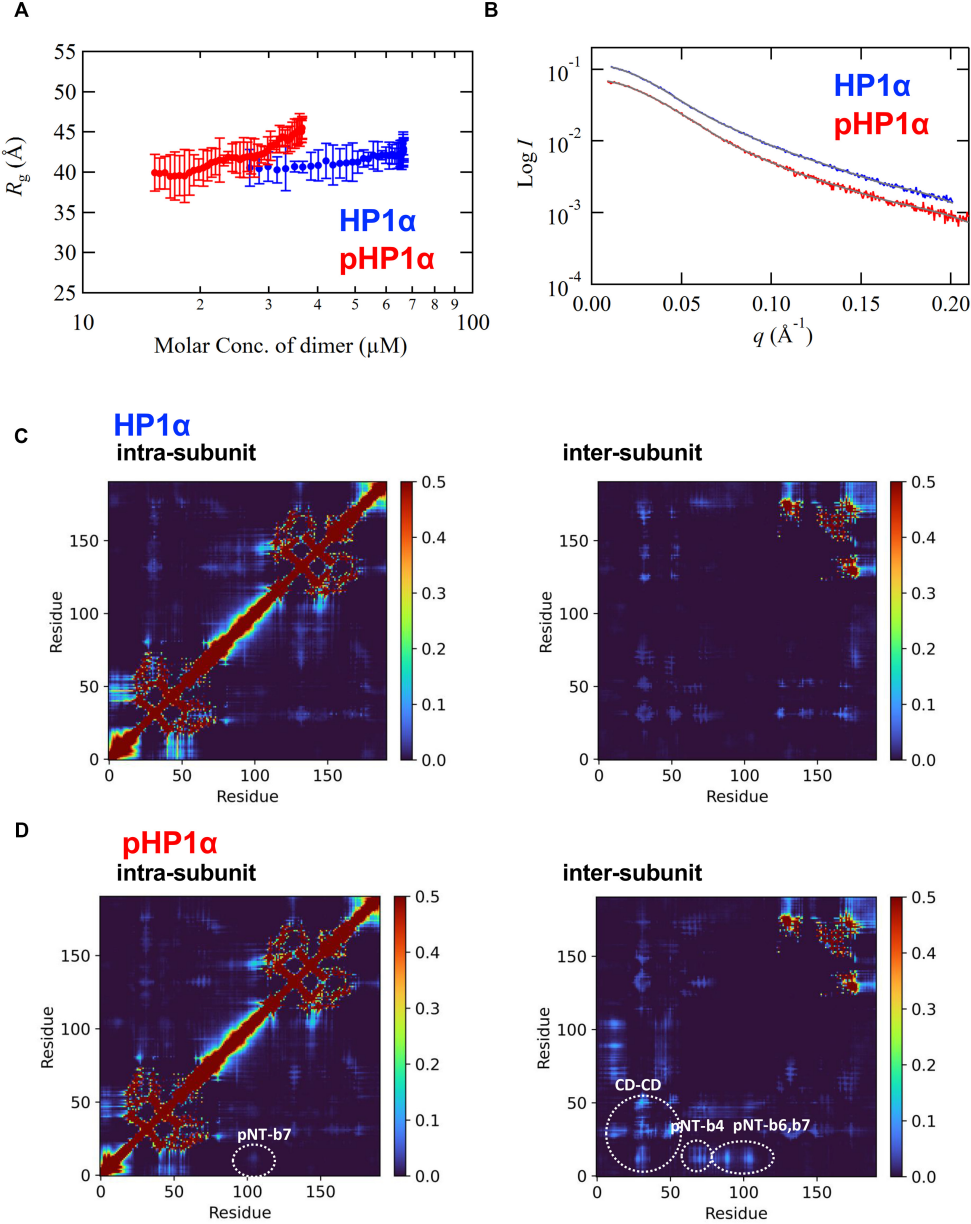
Comparison of phosphorylated and unphosphorylated HP1α by SAXS. (**A**) *R*_g_ values (see Supplementary Fig. S4A and B) plotted against molar concentration of dimer for HP1α and pHP1α at 50 mM NaCl. (**B**) SAXS profiles obtained by SEC-SAXS for HP1α and pHP1α at 50 mM NaCl. The lines represent the fits for both profiles obtained from EOM calculations (see Supplementary Fig. S4F). (**C** and **D**) Heatmaps of residue–residue interaction probabilities of HP1α (C) and pHP1α (D), calculated from the reweighted ensembles from CGMD simulations. Left and right panels show, respectively, intra- and inter-subunit residue–residue interactions, respectively.

The SEC-SAXS results for HP1α and pHP1α at 50 mM NaCl are shown in Supplementary Fig. S4A and B. These *I*(0) chromatograms showed not only that the peak broadening was more apparent for pHP1α than for HP1α but also that the *R*_g_ values around the peak were varied for both. Because the SEC-MALS data indicated that both HP1α and pHP1α were dimers, we plotted the *R*_g_ values against the dimer concentration (Fig. 2A), which showed that the *R*_g_ values of HP1α remained unchanged at concentrations below 40 μM, whereas those of pHP1α showed a concentration-dependent increase above ∼20 μM. This finding implies that dimer molecules with different conformational states due to the IDR region were present in the solution, and that electrostatic interactions between dimeric units have a more pronounced effect on pHP1α than on HP1α.

The experimental averaged-SAXS profiles for HP1α and pHP1α were derived from data in the respective concentration ranges shown in Fig. 2A, namely, 26.8−66.2 μM for the HP1α dimer and 15.3–36.4 μM for the pHP1α dimer (Fig. 2B). The *P*(*r*) functions calculated from these SAXS profiles (Supplementary Fig. S4E) suggest that, as an average depiction in solution, pHP1α has a core conformation that is roughly similar to that of HP1α, but also has a larger elongated domain overall. This trend seems to be consistent with that reported previously, albeit at different sample concentrations [20]. In order to explore the conformational ensemble of both molecules in solution, given the IDR regions in HP1α, we conducted EOM analysis on these experimental SAXS profiles and the dimer models to derive distributions of *D*_max_ (Fig. 2B and Supplementary Fig. S4F). In terms of the distribution, for HP1α, most molecules seemed to stay in the range of 100−170 Å, with *D*_max_ centered ∼130 Å. On the other hand, the *D*_max_ distribution of pHP1α showed a shift to a relatively smaller size (90−140 Å) and a slightly narrower peak width; conversely, however, it also showed that more extended structures >170 Å were present than observed in HP1α. Collectively, these observation indicate that two conformational states, a compact structure and an expanded structure, are generated in pHP1α in solution, suggesting the effect of various electrostatic interactions due to phosphorylation.

The *R*_g_ values of HP1α and pHP1α at 500 mM NaCl were larger than the corresponding values at 50 mM NaCl, suggesting an expansion of the overall structures of HP1α and pHP1α at high salt, due to reduced electrostatic intramolecular interactions (Supplementary Fig. S4C, D, and G). Furthermore, the *R*_g_ values of HP1α and pHP1α at 500 mM NaCl were not only comparable but also unchanged at around the peak, consistent with the NMR results, which showed similar chemical shifts for amino acids of HP1α and pHP1α at 500 mM NaCl.

In the HP1α and pHP1α dimers, there are two possible interactions among the monomers: intra-subunit and inter-subunit. To characterize the interactions in HP1α and pHP1α, we generated conformational ensembles using CGMD simulations to carry out a combined-analysis with SAXS (CGMD-SAXS). We performed five 5-μs simulations using a modified MARTINI force field [56, 57]. A theoretical SAXS profile was calculated for each snapshot of the simulations, and the snapshots were then reweighted by BME [65] so that the averaged SAXS profile could be fit to the experimental profile. The theoretical SAXS profiles calculated from the reweighted ensembles were well fitted to the experimental SAXS profiles for both HP1α and pHP1α (Supplementary Fig. S5A and B). The distribution plot of *D*_max_ calculated from reweighted ensembles (Supplementary Fig. S5C) was similar to the results obtained by EOM analysis of the experimental profiles (Supplementary Fig. S4F). The probability of interactions was calculated for each residue pair of the dimer in the reweighted ensembles (Fig. 2C and D); here, a pair of residues was defined as interacting when a CG bead of one residue was within 11 Å of that of the other residue.

In HP1α, there was a high frequency of intra-subunit interactions between the following acidic and basic segments (domain name in parentheses), a5(CSD)–b7(HR), suggesting that HR interacts well with CSD in the same subunit (Fig. 2C). In pHP1α, by contrast, there was a high probability of intra- and inter-subunit interactions between the phosphorylated N-tail and the basic segments b4(CD), b6(HR), and b7(HR) (Fig. 2D).

**Figure 3.**
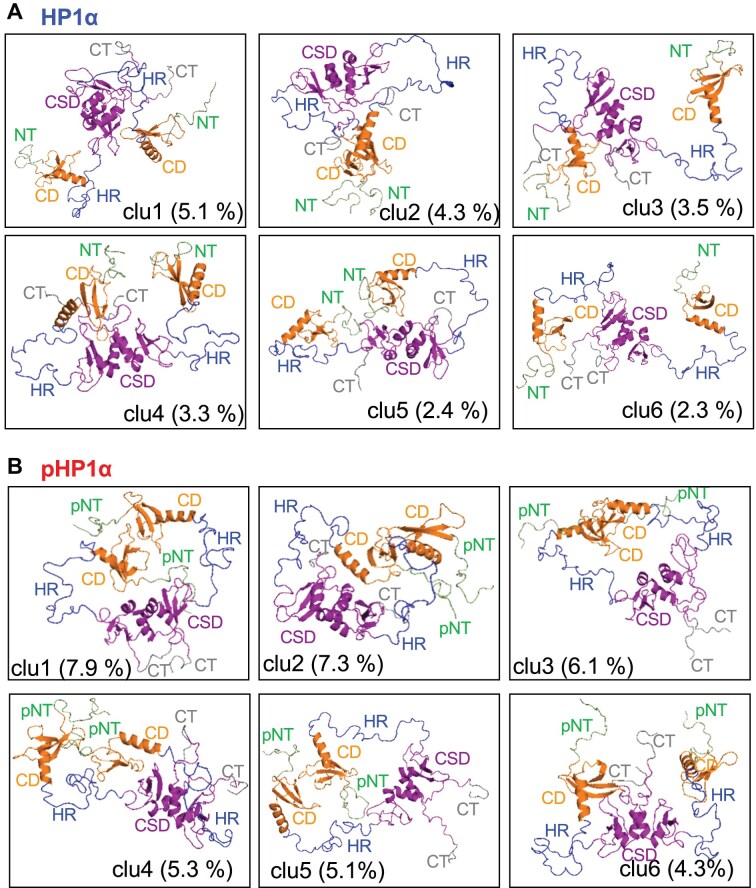
Differences in intramolecular interactions between phosphorylated and unphosphorylated HP1α. (**A** and **B**) Representative structures of the top six clusters from the reweighted ensembles of HP1α (A) and pHP1α (B). NT, pNT, and CT represent the N-tail, phosphorylated N-tail, and C-tail respectively.

The structures in the reweighted ensemble were classified into clusters by using a modified GROMOS algorithm, wherein the sum of the weights of neighbors was used instead of the number of neighbors to select the largest cluster (Fig. 3, and Supplementary Figs S6 and S7). The Cα-RMSD calculated for residues 11−14 (N-tail serines), 19−74 (CD), 89−91 (b6), 102−107 (b7), and 113−173 (CSD) after superimposing CSDs was used as the distance measure of the clustering, and the cutoff distance was set to 20 Å. Interestingly, most interactions occurred within the subunits of HP1α (Figs 2C and 3A, and Supplementary Fig. S6); however, in addition to the intra-subunit interaction of the phosphorylated N-tail with b7, pHP1α showed inter-subunit interactions as indicated by the CD–CD interactions (Fig. 2D) and by the phosphorylated N-tail interactions with b4 located at the end of CD and b6 and b7 in HR in almost all clusters except cluster 6 (Figs 2D and 3B, and Supplementary Fig. S7).

### Intramolecular interactions in CSD deletion mutants of HP1α and pHP1α revealed by NMR

To simplify the interactions of HP1α, we removed CSD, which is responsible for dimer formation and intra- and inter-subunit interactions as described above. The CSD deletion mutant of HP1α_S97A, designated as ΔCSD, is intrinsically a monomer due to the lack of CSD.

First, we observed ^1^H-^15^N HSQC spectra of ΔCSD and phosphorylated ΔCSD (pΔCSD) at 120 μM (Supplementary Fig. S8A). Although no secondary structural differences between pHP1α and pΔCSD were found (Supplementary Fig. S8B), small chemical shift differences were observed in individual residues between pHP1α and pΔCSD, and between HP1α and ΔCSD (Supplementary Fig. S8C).

**Figure 4.**
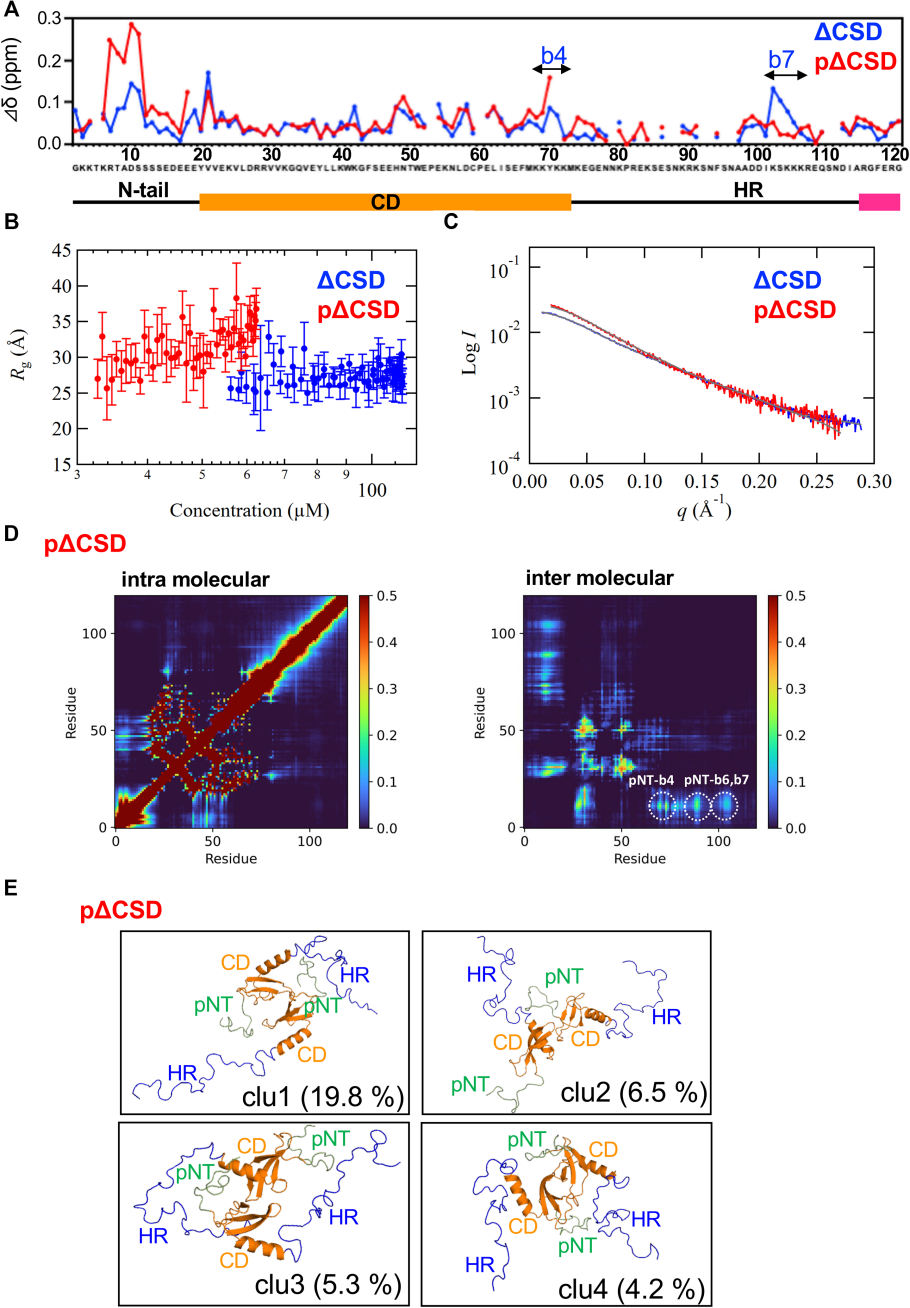
Comparison of phosphorylated and unphosphorylated ΔCSD by NMR and SAXS. (**A**) Differences in chemical shift between 500 and 50 mM NaCl. (**B**) *R*_g_ values (see Supplementary Fig. S9C and D) plotted against molar concentration of monomer for ΔCSD and pΔCSD. (**C**) SAXS profiles obtained by SEC-SAXS for ΔCSD and pΔCSD at 50 mM NaCl. The lines represent the fits for both profiles obtained from EOM calculations (see Supplementary Fig. S9F). (**D**) Heatmaps of residue–residue interaction probabilities of pΔCSD calculated from the reweighted ensemble from the CGMD simulation of the two-molecule system. Left and right panels show, respectively, intra- and intermolecular residue–residue interactions. (**E**) Representative structures of the top four clusters from the reweighted ensemble of pΔCSD. pNT represents the phosphorylated N-tail.

Interestingly, similar chemical shift changes were observed in ΔCSD and pΔCSD between the 500 and 50 mM NaCl conditions, as observed for HP1α and pHP1α (Fig. 4A). This suggests that, while HP1α and pHP1α both showed chemical shift changes in their CSD and the C-terminal regions, electrostatic interactions mainly exist in their N-terminal, CD, and HR regions. In particular, ΔCSD showed marked chemical shift changes in b7 (Lys102–Lys105), as observed in HP1α.

In addition, significant differences observed between HP1α and pHP1α were similarly identified between ΔCSD and pΔCSD for Tyr20; Lys42; the segment of His 48, Asn49, Thr50, and Trp41 after a2; the segment of Glu54–Cys59; and Tyr70 (Fig. 4A). Again, the residues in CD of ΔCSD showing significant chemical shift changes are located near the aromatic cage (Tyr20, Trp41, and Phe44) required for H3K9me_3_ binding.

### Overall structures of ΔCSD and pΔCSD revealed by SEC-MALS/SAXS and CGMD-SAXS

Next, we analyzed the overall structures and characteristics of the CSD deletion mutants, ΔCSD and pΔCSD, at 50 mM NaCl using SEC-MALS/SAXS and EOM analysis (Fig. 4B and Supplementary Fig. S9). The molar mass values obtained by SEC-MALS were 16.3 and 14.9 kDa, respectively, suggesting that both ΔCSD and pΔCSD are in the monomeric form (Supplementary Fig. S9A and B). As shown in Fig. 4B, ΔCSD showed little variation in *R*_g_ over the concentration range measured (56.1–113.3 μM), while a gradual increase in *R*_g_ was observed for pΔCSD over a slightly lower concentration range (32.6–62.3 μM). This trend was similar to that observed for HP1α/pHP1α, indicating that, even for the pΔCSD monomer, the structure is affected by intermolecular interactions arising from small steps in concentration (Fig. 4B). The experimental averaged-SAXS profiles for ΔCSD and pΔCSD derived from data in these concentration ranges are shown in Fig. 4C. The *P*(*r*) functions calculated from these SAXS profiles (Supplementary Fig. S9E) suggesting that, as an average depiction in solution, pΔCSD has a slightly broadened conformation as compared with ΔCSD. On the other hand, EOM analysis with the monomer models showed that ΔCSD and pΔCSD have diverse structural ensembles, while the distribution of *D*_max_ suggested that pΔCSD has a larger proportion of compact structures as compared with ΔCSD (Supplementary Fig. S9F), similar to the findings for HP1α and pHP1α. This may be due to the effect of intramolecular electrostatic interactions between the phosphorylated N-tail and the basic segment, as also observed in the NMR data (Fig. 4A), but is not consistent with the average depiction calculated by the *P*(*r*) function.

To further characterize the molecular structure and interactions within ΔCSD and pΔCSD, we performed CGMD-SAXS analysis. CGMD simulations of ΔCSD and pΔCSD were performed in single-molecule and two-molecule systems, and the ensembles obtained were reweighted by BME in order to reproduce the experimental SAXS profiles. For ΔCSD, the SAXS profile calculated with the reweighted ensemble from the single-molecule system was fitted to the experimental results better than the one calculated with the reweighted ensemble from the two-molecule system, suggesting that ΔCSD exists as a monomer (Supplementary Fig. S10A and B). For pΔCSD, by contrast, the SAXS profile calculated with the reweighted ensemble from the two-molecule system showed better agreement with the experimental profile (Supplementary Fig. S10C and D), suggesting that pΔCSD forms a dynamic dimer.

Next, residue–residue interaction probability calculations and cluster analysis were performed for the reweighted ensemble of the two-molecule system of pΔCSD (Fig. 4D). The Cα-RMSD calculated for residues 11−14 (N-tail serines), 19−74 (CD), 89−91 (b6), and 102−107 (b7) of both molecules was used as the distance measure of the clustering and the cutoff distance was set to 10 Å. The interaction probability map suggested the presence of intermolecular interactions between the phosphorylated N-tail in one molecule and basic segments b4(CD), b6(HR), and b7(HR) in the other molecule (Fig. 4D, right). Representative structures of the top clusters are shown in Fig. 4E and Supplementary Fig. S11; the top three clusters showed intermolecular interactions between pΔCSD and other pΔCSD molecules (Fig. 4E and Supplementary Fig. S11). Overall, the chemical shift changes of pΔCSD in the NMR experiment (Fig. 4A) suggest that the phosphorylated N-tail has intermolecular interactions mainly with b4, in addition to intramolecular interactions.

**Figure 5.**
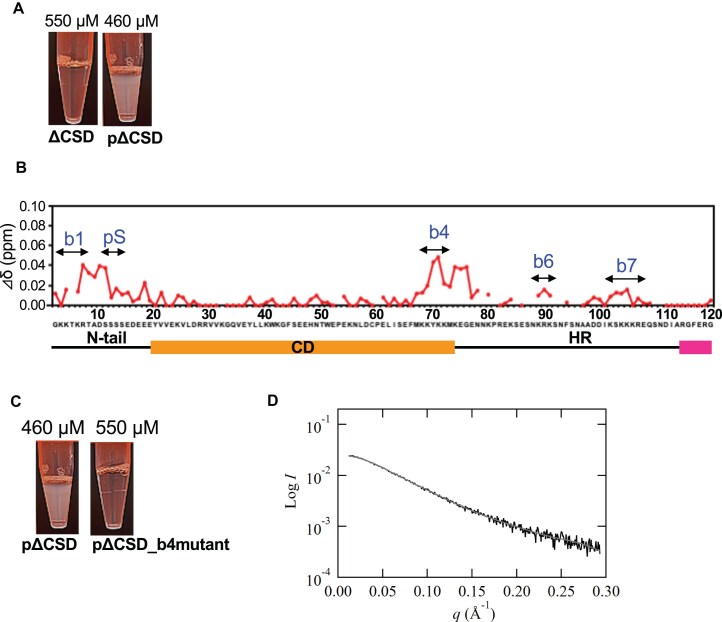
LLPS of the phosphorylated ΔCSD. (**A**) Difference in appearance of the condensed solution between ΔCSD and pΔCSD. (**B**) Chemical shift differences (Δδ) of pΔCSD between mid (120 μM) and condensed (400 μM) solutions at 50 mM NaCl. (**C**) Difference in appearance of the condensed solution between pΔCSD and the pΔCSD_b4 mutant. (**D**) SAXS profile for the pΔCSD_b4 mutant at 50 mM NaCl (black). The gray fitted line is derived from the EOM calculation (see Supplementary Fig. S15E).

Because pΔCSD can undergo LLPS like pHP1α (Fig. 5A, and Supplementary Fig. S12C and D), we compared the NMR spectra of pΔCSD at 120 and 400 μM to mimic the situation before and after LLPS (Supplementary Fig. S13A). Significant signal changes were observed for the basic segments b4, b6, and b7, and the phosphorylated N-tail (Fig. 5B). In particular, large chemical shift differences were observed for residues in the phosphorylated N-tail and the basic segment b4(CD) (Fig. 5B). In good agreement with the CGMD-SAXS data (Fig. 4D and E), the basic segment b4(CD) was found to interact with the phosphorylated N-tail of another molecule. These results suggest that the phosphorylated N-tail and CD are important for intermolecular interactions of the pHP1α for LLPS conditions.

Because the basic segment b4(CD) may play a key role in LLPS, we therefore constructed a phosphorylated ΔCSD_b4 mutant (pΔCSD_b4) in which the Lys68–Lys72 residues were each replaced with alanine. There were no structural alterations due to the b4 mutation, and the α-helix at the end of the CD became stabilized (Supplementary Fig. S14). Increasing the concentration of pΔCSD_b4 mutant did not cause LLPS (Fig. 5C and Supplementary Fig. S12D), suggesting that the b4(CD) segment of pHP1α is important in the intermolecular interactions required for LLPS.

SEC-MALS/SAXS measurements of pΔCSD_b4 were also performed at 50 mM NaCl (Supplementary Fig. S15). The molar mass obtained from SEC-MALS was 13.7 kDa, indicating that pΔCSD_b4 was a monomer (Supplementary Fig. S15A). In SEC-SAXS analysis in the concentration range 32.2–67.5 μM, no change in *R*_g_ was observed, different with the results of pΔCSD over a similar concentration range (Supplementary Fig. S15C). The experimental averaged-SAXS profiles for pΔCSD_b4, derived from data in the above concentration range, is shown in Fig. 5D. Regarding the *P*(*r*) function calculated from the SAXS profile (Supplementary Fig. S15D), the function shape of pΔCSD_b4 was quite similar to that of ΔCSD and the two *D*_max_ values were consistent. In EOM analysis, the distribution of *D*_max_ for pΔCSD_b4 was larger than that for pΔCSD and similar to that of ΔCSD (Supplementary Fig. S15E).

**Figure 6.**
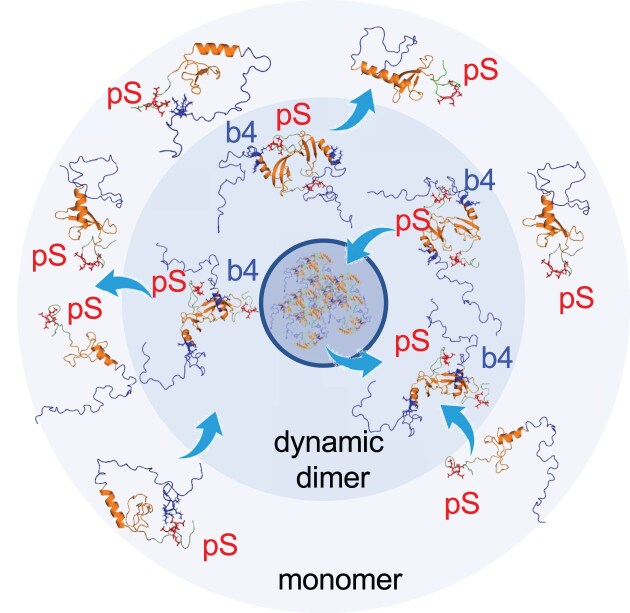
Model of LLPS of the phosphorylated ΔCSD. LLPS is mediated by dynamic intermolecular dimers formed via the phosphorylated N-tail (pS) and an essential basic segment located at the end of CD (b4).

We combined the results of NMR, SEC-MALS/SAXS, and CGMD-SAXS analysis to construct the following model of LLPS (Fig. 6). In the low concentration condition, dynamic interactions between the phosphorylated N-tails and basic segments (especially b7) of pΔCSD are limited to intramolecular interactions, leading to a compact conformation. As the concentration increases, intermolecular interactions of pΔCSD initially form a dynamic dimer, which is observable by NMR as a core structural unit of LLPS existing in solutions outside droplets; the dynamic dimer then forms multimeric oligomers causing droplets, which could not be detected by NMR (Fig. 6). In particular, the interaction between b4(CD) and the phosphorylated N-tail is important for the intermolecular interactions involved in LLPS (Fig. 6).

### Effect of b4 mutation on the heterochromatic localization

**Figure 7.**
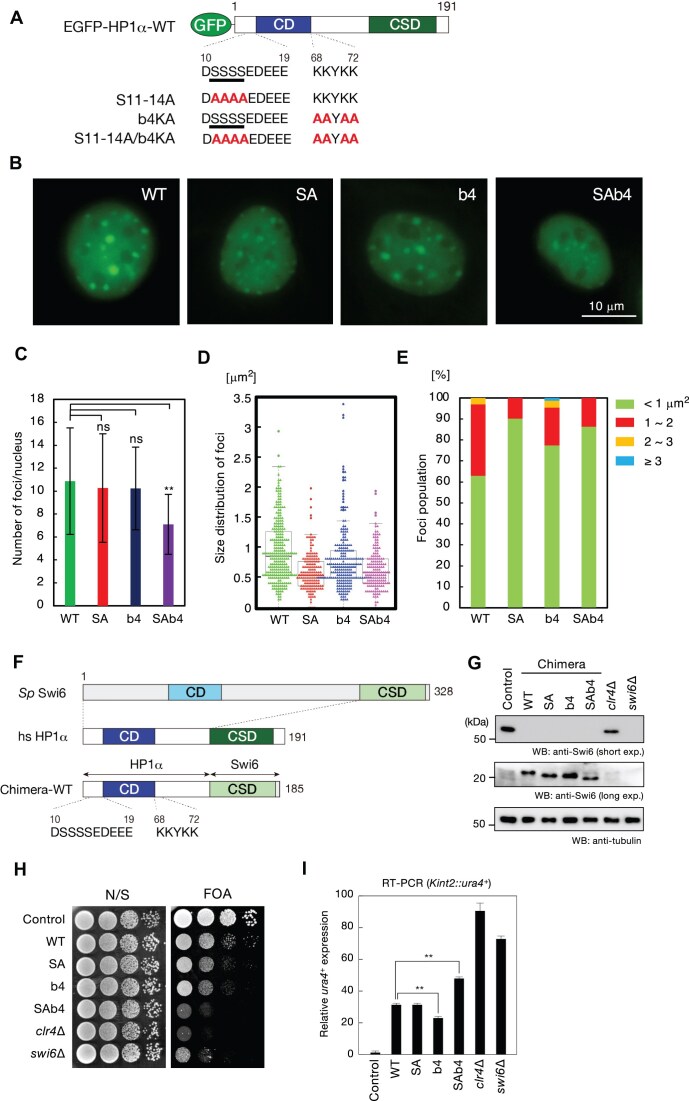
Effect of b4 mutation on heterochromatic localization of HP1α. (**A**) Schematic diagram of EGFP-fused HP1α showing the amino acid sequence of the N-terminal region containing the phosphorylation sites (S11–S14) and the b4 segment (K68–K72). Serine residues that can be phosphorylated are underlined; mutated amino acid residues are indicated in bold; two boxes in HP1α represent the conserved CD and CSD, respectively. (**B**) Example images of NIH3T3 cells transfected with EGFP-fused WT or mutant (SA, b4, or SAb4) HP1α. Scale bar: 10 μm. (**C**) Number of HP1α foci detected in the nucleus of transfected cells. Statistical significance of differences relative to WT was determined by Mann–Whitney’s U test. ns, not satisfied; **P < 0.01. (**D**) Size of HP1α foci detected in the nucleus of transfected cells calculated and shown by beeswarm plot. (**E**) Percentages of HP1α foci classified in accordance with size (<1 μm^2^; ≥1 and < 2 μm^2^; ≥2 and < 3 μm^2^; ≥3 μm^2^). (**F**) Schematic diagram of *Schizosaccharomyces pombe* (*Sp*) Swi6, human (hs) HP1α, and a chimeric protein (Chimera-WT) containing HP1α NCD and Swi6 CSD showing the amino acid sequences of the N-terminal region containing the phosphorylation sites (S11–S14) and the b4 segment (K68–K72). (**G**) Immunoblotting analysis of WT Swi6 and chimeric HP1α/Swi6 proteins. Whole-cell extracts prepared from WT cells (control) or cells expressing WT or mutant chimeric proteins were subjected to immunoblotting using anti-Swi6 antibody raised against full-length Swi6 protein. Due to the lower reactivity of chimeric proteins, a longer exposure image is shown to confirm expression. Anti-tubulin antibody was used as a control. (**H**) Spotting assays for *Kint2*::*ura4*^+^ silencing. A serially diluted culture of the indicated strains was spotted onto nonselective medium (N/S) or medium containing 5FOA (FOA). (**I**) Expression of the *ura4*^+^ silencing reporter evaluated by quantitative RT-PCR analysis. Results are means ± s.d. of at least three independent experiments. Statistical significance relative to WT was determined by Mann–Whitney’s *U* test; **P < 0.01.

To probe the physiological role of the b4 segment of pHP1α, WT or mutant HP1α was transiently expressed as an EGFP fusion protein in NIH3T3 cells, and its colocalization with the heterochromatic region was examined by fluorescence microscopy. Three mutations were introduced into HP1α: S11–14A mutation in the N-terminal phosphorylation site (SA mutant); b4KA mutation in the basic segment of CD (b4 mutant); and mutations in both S11-14A and b4KA (SAb4 mutant) (Fig. 7A). As previously observed [16, 70], EGFP-fused WT HP1α showed punctate nuclear signals corresponding to heterochromatic regions (Fig. 7B). The b4 mutant also showed punctate nuclear signals and colocalized with the heterochromatic regions (Fig. 7B). The SA and SAb4 mutants both showed a marked decrease in the cell population with clear heterochromatic localization (Fig. 7B). We quantified the number and size of foci showing heterochromatin localization of the HP1α variants, and the frequency of foci by size (Fig. 7C–E). There was no significant difference in the number of HP1α foci among the SA and b4 mutants and WT; however, the number of foci was reduced in the SAb4 double mutant (Fig. 7C). The overall size of the HP1α foci was reduced in the SA mutant as compared with WT, and the size of the HP1α foci was slightly reduced in the b4 mutant, but there was a high degree of variability; however, the size of the HP1α foci was clearly reduced in the SAb4 double mutant (Fig. 7D). The population with a size >1 μm^2^ was clearly smaller in the SA mutant than in WT, and was also smaller in the b4 mutant. All HP1α foci in the SA mutant and the SAb4 double mutant were <2 μm^2^, while HP1α foci <1 μm^2^ accounted for nearly 90% of foci in the Sab4 double mutant (Fig. 7E). Collectively these observations suggest that phosphorylation of the N-terminal is involved in the maintenance or fusion of HP1α foci, but not in the initiation of HP1 foci formation, while the b4 segment functions in regulation of the fusion of HP1 foci. The SAb4 double mutant might also have an effect on HP1α foci formation.

In fission yeast, Swi6, one of two HP1 proteins, plays a major role in heterochromatin formation and forms stable dimer via its CSD, which also mediates interactions with other transcription factors. To examine the physiological importance of CSD of HP1α, we therefore used fission yeast as a model and assessed the effect of different mutations on heterochromatic silencing. Chimeric proteins containing the N-tail CD of HP1α and the CSD of Swi6 (Chimera-WT) were expressed from the endogenous *swi6* locus (Fig. 7F and G), and their silencing ability was assessed by monitoring the expression of a reporter gene inserted in the mating-type *K* region (*Kint2::ura4*^+^). Cells were cultured, serially diluted, and spotted onto either non-selective medium or medium containing FOA (toxic to *ura4*^+^-expressing cells).

We found that the *ura4*^+^ gene inserted into the mating-type region was tightly repressed in WT cells, which grew well on FOA plates (Fig. 7H), whereas a lack of Swi6 or Clr4 (H3K9 histone methyltransferase) led to derepression of the reporter gene, as evidenced by no or poor growth on the FOA medium (Fig. 7H). The derepression status of *Kint2::ura4*^+^ was confirmed by quantitative RT-PCR analysis (Fig. 7G). While *Kint2::ura4*^+^ showed partial derepression in cells expressing Chimera-WT (Fig. 7G), it robustly grew on FOA medium (Fig. 7H), indicating that it maintains silencing function. We also examined the silencing function of mutant chimeric proteins containing the SA, b4, or SAb4 mutation (Fig. 7G–I). While the SA or b4 single mutation mildly affected the silencing function of the chimeric protein, the SAb4 double mutation led to a clear silencing defect (Fig. 7H and I), suggesting that the phosphorylated serine cluster and b4 segment cooperatively function to assemble silent heterochromatin *in vivo*.

## Discussion

Herein, we have examined the intra- and intermolecular interactions of HP1α, the N-terminal pHP1α, the CSD deletion mutant (ΔCSD), and its phosphorylated form (pΔCSD) by using a combination of NMR, SEC-MALS/SAXS, and CGMD-SAXS methods to probe the proteins at their appropriate concentrations. Our experiments, supported by simulations, have identified a number of interactions that contribute to LLPS of pHP1α. First, NMR and SEC-MALS revealed that pHP1α forms a dimer at a concentration of ∼150 μM and makes inter-subunit interactions via the phosphorylated N-tail and the basic segments b4, b6, and b7 (Figs 1C and 2D), indicating that phosphorylation of the N-tail of HP1α facilitates the electrostatic interactions. This finding is well correlated with earlier findings on the role of phosphorylation in phase separation [20, 23]. A previous MD simulation also showed that, in unphosphorylated HP1α, the basic segments of b4, b6, and b7 interact with DNA [71], and these segments are necessary for selective binding of HP1α to the nucleosome containing trimethylated histone H3 [72]. These observations suggest that nucleosomal DNA inhibits the interaction of the phosphorylated N-tail of pHP1α with these basic segments, enabling the free phosphorylated N-tail to interact with the basic segment of the H3 N-tail after H3 trimethylation, as has been previously shown for the N-terminal fragment consisting of just the N-tail and CD [17].

We also found that the overall structure of pHP1α at ∼40 μM is much more compact than that of HP1α, as demonstrated by SEC-SAXS (Supplementary Fig. S4F). This indicates that multi-electrostatic interactions between the basic segments b4, b6, and b7 and the phosphorylated N-tail are responsible for the compact conformations of pHP1α. At a concentration of ∼150 μM, pHP1α formed droplets (Supplementary Fig. S12B), while at concentrations of 30 μM and higher, it tended to form multimeric oligomers, as shown by SEC-SAXS (Fig. 2A). In contrast, SEC-MALS indicated that both HP1α and pHP1α formed stable dimers at similar concentrations. Collectively, these observations suggest that at a concentration of ∼150 μM pHP1α exists mainly as a stable dimer, which dynamically forms multimeric oligomers causing droplets; at this concentration, however, NMR signals from the dimer are perturbed by dynamic equilibrium with multimeric oligomers. These results are consistent with a recent CG simulation study showing that pHP1α adopts a compact form compared with HP1α and forms liquid droplets at concentrations >50 μM [23, 25].

At higher concentrations of about 500 μM, the NMR signal intensities of the pHP1α dimer decreased sharply, especially in the CSD region (Supplementary Fig. S18), indicating multimeric oligomer formation of the pHP1α via CSD. As a result, we could not use NMR to obtain detailed information on the interaction modes of pHP1α responsible for LLPS formation. However, the NMR spectrum of pΔCSD in the LLPS state provided molecular details of the intermolecular interactions between the phosphorylated N-tail and the basic segment b4 located at the end of CD. A previous CG simulation suggested that CD is more critical than CSD in influencing the intermolecular interactions that drive phase separation [32], which supports our CSD deletion experiment in revealing the structural unit of pHP1α in LLPS.

NMR together with SEC-MALS/SAXS experiments suggested that, at a concentration of ∼120 μM, pΔCSD was in equilibrium between the monomer and dimer. Furthermore, at higher concentrations (120–400 μM), pΔCSD underwent increased droplet formation (Supplementary Fig. S12); however, the intensities of NMR signals of pΔCSD were almost identical at 120 and 400 μM (Supplementary Fig. S13B). These results suggest that the NMR signals did not derive from the droplets themselves, but from the dynamic dimer–dimer interactions of pΔCSD in solution outside the droplets. As the core structural unit of LLPS, the dynamic dimer of pΔCSD is responsible for droplet formation, which occurs as the concentration of pΔCSD increases from 120 to 400 μM and the dimers undergo multimeric oligomerization (Fig. 6). In the case of full-length pHP1α interactions with neighboring pHP1α dimers produce an oligomer with a total molecular weight of ∼140 kDa, making it difficult to observe the NMR signals of the interacting pHP1α dimer. By contrast, the molecular weight of the pΔCSD dimer is ∼30 kDa; thus, it was possible to observe dynamic dimer–dimer interactions of pΔCSD. Our SAXS experiments also showed slight increases in Rg values with pΔCSD concentration (Figs 2A and 4B), indicative of dimer–dimer interactions. In addition, CGMD simulations reproduced the dynamic equilibrium between the monomers and dimers of pΔCSD, whereby the phosphorylated N-tail interacts mainly with b4 (Fig. 4D). Combining the NMR, SAXS, and CGMD simulation results, we further showed that pΔCSD at low concentration adopts the monomer conformation with intramolecular interactions between the phosphorylated N-tail and mainly b4 and then b6 or b7; and that, upon increasing concentration, pΔCSD dynamically forms dimers via the phosphorylated N-tail and mainly b4 or b6 and then b7. In the case of pΔCSD, the dynamic dimer is likely to be the core structural unit for forming LLPS, enabling us to successfully capture the dynamic intermediate in LLPS (Fig. 6).

Previously, alanine replacement of the basic residues in b6 of pHP1α impaired LLPS ability *in vitro* [20]. Here, we found that the basic segment b4 is also essential for pHP1α to undergo LLPS *in vitro*. Our *in vivo* heterochromatic localization experiment further showed that b4 plays an important role in the correct formation of HP1α foci (Fig. 7), which is well correlated with our observation that the pΔCSD_b4 mutant has impaired LLPS ability (Fig. 5C) and with a previous study showing that the puncta size of pHP1α is larger than that of HP1α in NIH3 cells expressing Cy3-labeled HP1 and pHP1 [20]. We also found that unphosphorylated HP1α led to a smaller foci size than pHP1α and that mutation of b4 led to changes in the size of HP1α foci.

In summary, our integrative structural methods have identified the basic segment b4 located at the end of CD as central to the ability of HP1α to undergo LLPS. Among the three HP1 homologues in mammals, HP1α, HP1β, and HP1γ, only HP1α undergoes LLPS *in vitro* and the basic segment b4 is an HP1α specific sequence in the three homologs [73]. A previous hinge-swapping simulation demonstrated that the positively charged lysine/arginine residue clusters in the HP1α region of Lys68–Arg115 are necessary for intermolecular interaction [32]. The basic segment b4 (Lys68–Lys72) in our study is located at the end of CD (Tyr20–Met73), which is followed by HR. Because basic segments in the simulated region other than b4 are conserved in the three HP1 species, the previous simulation study [32] is in good agreement with our findings. DNA molecules are known to promote HP1α LLPS [21, 34], and recently nuclear RNA has been reported to promote the fusion of HP1α foci [74]. Thus, it seems likely that the basic segment b4 at the end of CD of HP1α interacts with DNA and RNA to form the correct foci for silencing heterochromatin in cells; however, further studies will be needed to reveal the different roles of HP1α, HP1β, and HP1γ *in vivo*.

## Supplementary Material

gkaf154_Supplemental_Files

## Data Availability

The chemical shifts have been deposited in the Biological Magnetic Resonance Data Bank (BMRB) under accession number 52336. The structure information has been submitted to the Small-Angle-Scattering Biological Data Bank (SASBDB; https://www.sasbdb.org/aboutSASBDB/) [53] under the following IDs: SASBDB are SASDU23, SASDU33, SASDU43, SASDU53, SASDU63, SASDU73, and SASDU83.

## References

[1] Grewal SI, Elgin SC Heterochromatin: new possibilities for the inheritance of structure. Curr Opin Genet Dev. 2002; 12:178–87. 10.1016/S0959-437X(02)00284-8. 11893491

[2] Richards EJ, Elgin SC Epigenetic codes for heterochromatin formation and silencing: rounding up the usual suspects. Cell. 2002; 108:489–500. 10.1016/S0092-8674(02)00644-X. 11909520

[3] Grewal SI, Moazed D Heterochromatin and epigenetic control of gene expression. Science. 2003; 301:798–802. 10.1126/science.1086887. 12907790

[4] Bannister AJ, Zegerman P, Partridge JF et al. Selective recognition of methylated lysine 9 on histone H3 by the HP1 chromo domain. Nature. 2001; 410:120–4. 10.1038/35065138. 11242054

[5] Lachner M, O’Carroll D, Rea S et al. Methylation of histone H3 lysine 9 creates a binding site for HP1 proteins. Nature. 2001; 410:116–20. 10.1038/35065132. 11242053

[6] Nakayama J, Rice JC, Strahl BD et al. Role of histone H3 lysine 9 methylation in epigenetic control of heterochromatin assembly. Science. 2001; 292:110–3. 10.1126/science.1060118. 11283354

[7] Nielsen AL, Oulad-Abdelghani M, Ortiz JA et al. Heterochromatin formation in mammalian cells: interaction between histones and HP1 proteins. Mol Cell. 2001; 7:729–39. 10.1016/S1097-2765(01)00218-0. 11336697

[8] Jacobs SA, Khorasanizadeh S Structure of HP1 chromodomain bound to a lysine 9-methylated histone H3 tail. Science. 2002; 295:2080–3. 10.1126/science.1069473. 11859155

[9] Jacobs SA, Taverna SD, Zhang Y et al. Specificity of the HP1 chromo domain for the methylated N-terminus of histone H3. EMBO J. 2001; 20:5232–41. 10.1093/emboj/20.18.5232. 11566886 PMC125272

[10] Nielsen PR, Nietlispach D, Mott HR et al. Structure of the HP1 chromodomain bound to histone H3 methylated at lysine 9. Nature. 2002; 416:103–7. 10.1038/nature722. 11882902

[11] Thiru A, Nietlispach D, Mott HR et al. Structural basis of HP1/PXVXL motif peptide interactions and HP1 localisation to heterochromatin. EMBO J. 2004; 23:489–99. 10.1038/sj.emboj.7600088. 14765118 PMC1271814

[12] Azzaz AM, Vitalini MW, Thomas AS et al. Human heterochromatin protein 1alpha promotes nucleosome associations that drive chromatin condensation. J Biol Chem. 2014; 289:6850–61. 10.1074/jbc.M113.512137. 24415761 PMC3945347

[13] Nishibuchi G, Nakayama J Biochemical and structural properties of heterochromatin protein 1: understanding its role in chromatin assembly. J Biochem. 2014; 156:11–20. 10.1093/jb/mvu032. 24825911

[14] Canzio D, Chang EY, Shankar S et al. Chromodomain-mediated oligomerization of HP1 suggests a nucleosome-bridging mechanism for heterochromatin assembly. Mol Cell. 2011; 41:67–81. 10.1016/j.molcel.2010.12.016. 21211724 PMC3752404

[15] Maison C, Almouzni G HP1 and the dynamics of heterochromatin maintenance. Nat Rev Mol Cell Biol. 2004; 5:296–305. 10.1038/nrm1355. 15071554

[16] Hiragami-Hamada K, Shinmyozu K, Hamada D et al. N-terminal phosphorylation of HP1alpha promotes its chromatin binding. Mol Cell Biol. 2011; 31:1186–200. 10.1128/MCB.01012-10. 21245376 PMC3067897

[17] Shimojo H, Kawaguchi A, Oda T et al. Extended string-like binding of the phosphorylated HP1alpha N-terminal tail to the lysine 9-methylated histone H3 tail. Sci Rep. 2016; 6:22527 10.1038/srep22527. 26934956 PMC4776139

[18] Nishibuchi G, Machida S, Osakabe A et al. N-terminal phosphorylation of HP1alpha increases its nucleosome-binding specificity. Nucleic Acids Res. 2014; 42:12498–511. 10.1093/nar/gku995. 25332400 PMC4227797

[19] Machida S, Takizawa Y, Ishimaru M et al. Structural basis of heterochromatin formation by Human HP1. Mol Cell. 2018; 69:385–97. 10.1016/j.molcel.2017.12.011. 29336876

[20] Larson AG, Elnatan D, Keenen MM et al. Liquid droplet formation by HP1alpha suggests a role for phase separation in heterochromatin. Nature. 2017; 547:236–40. 10.1038/nature22822. 28636604 PMC5606208

[21] Strom AR, Emelyanov AV, Mir M et al. Phase separation drives heterochromatin domain formation. Nature. 2017; 547:241–5. 10.1038/nature22989. 28636597 PMC6022742

[22] Sanulli S, Trnka MJ, Dharmarajan V et al. HP1 reshapes nucleosome core to promote phase separation of heterochromatin. Nature. 2019; 575:390–4. 10.1038/s41586-019-1669-2. 31618757 PMC7039410

[23] Her C, Phan TM, Jovic N et al. Molecular interactions underlying the phase separation of HP1alpha: role of phosphorylation, ligand and nucleic acid binding. Nucleic Acids Res. 2022; 50:12702–22. 10.1093/nar/gkac1194. 36537242 PMC9825191

[24] Guthmann M, Qian C, Gialdini I et al. A change in biophysical properties accompanies heterochromatin formation in mouse embryos. Genes Dev. 2023; 37:336–50. 10.1101/gad.350353.122. 37072228 PMC10153458

[25] Elathram N, Ackermann BE, Clark ET et al. Phosphorylated HP1alpha-nucleosome interactions in phase separated environments. J Am Chem Soc. 2023; 145:23994–4004. 10.1021/jacs.3c06481. 37870432 PMC10636758

[26] Erdel F, Rademacher A, Vlijm R et al. Mouse heterochromatin adopts digital compaction states without showing hallmarks of HP1-driven liquid–liquid phase separation. Mol Cell. 2020; 78:236–49. 10.1016/j.molcel.2020.02.005. 32101700 PMC7163299

[27] Singh PB, Newman AG HP1-Driven micro-phase separation of heterochromatin-like domains/complexes. Genet Epigenet. 2022; 15:25168657221109766 10.1177/25168657221109766. PMC9260563 35813402

[28] Nozaki T, Imai R, Tanbo M et al. Dynamic organization of chromatin domains revealed by super-resolution live-cell imaging. Mol Cell. 2017; 67:282–93. 10.1016/j.molcel.2017.06.018. 28712725

[29] Gibson BA, Doolittle LK, Schneider MWG et al. Organization of chromatin by intrinsic and regulated phase separation. Cell. 2019; 179:470–84. 10.1016/j.cell.2019.08.037. 31543265 PMC6778041

[30] Laflamme G, Mekhail K Biomolecular condensates as arbiters of biochemical reactions inside the nucleus. Commun Biol. 2020; 3:773 10.1038/s42003-020-01517-9. 33319830 PMC7738674

[31] Zhang H, Qin W, Romero H et al. Heterochromatin organization and phase separation. Nucleus. 2023; 14:2159142 10.1080/19491034.2022.2159142. 36710442 PMC9891170

[32] Phan TM, Kim YC, Debelouchina GT et al. Interplay between charge distribution and DNA in shaping HP1 paralog phase separation and localization. eLife. 2024; 12:e90820 10.7554/eLife.90820.3. PMC11003746 38592759

[33] Tortora MMC, Brennan LD, Karpen G et al. HP1-driven phase separation recapitulates the thermodynamics and kinetics of heterochromatin condensate formation. Proc Natl Acad Sci USA. 2023; 120:e2211855120 10.1073/pnas.2211855120. 37549295 PMC10438847

[34] Keenen MM, Brown D, Brennan LD et al. HP1 proteins compact DNA into mechanically and positionally stable phase separated domains. eLife. 2021; 10:e64563 10.7554/eLife.64563. 33661100 PMC7932698

[35] Itoh Y, Woods EJ, Minami K et al. Liquid-like chromatin in the cell: what can we learn from imaging and computational modeling?. Curr Opin Struct Biol. 2021; 71:123–35. 10.1016/j.sbi.2021.06.004. 34303931

[36] Hansen JC, Maeshima K, Hendzel MJ The solid and liquid states of chromatin. Epigenetics Chromatin. 2021; 14:50 10.1186/s13072-021-00424-5. 34717733 PMC8557566

[37] Shin Y, Chang YC, Lee DSW et al. Liquid nuclear condensates mechanically sense and restructure the genome. Cell. 2018; 175:1481–91. 10.1016/j.cell.2018.10.057. 30500535 PMC6724728

[38] Vernon RM, Chong PA, Tsang B et al. Pi–Pi contacts are an overlooked protein feature relevant to phase separation. eLife. 2018; 7:e31486 10.7554/eLife.31486. 29424691 PMC5847340

[39] Boeynaems S, Alberti S, Fawzi NL et al. Protein phase separation: a new phase in cell biology. Trends Cell Biol. 2018; 28:420–35. 10.1016/j.tcb.2018.02.004. 29602697 PMC6034118

[40] Sawano A, Miyawaki A Directed evolution of green fluorescent protein by a new versatile PCR strategy for site-directed and semi-random mutagenesis. Nucleic Acids Res. 2000; 28:E78 10.1093/nar/28.16.e78. 10931937 PMC108465

[41] Delaglio F, Grzesiek S, Vuister GW et al. NMRPipe: a multidimensional spectral processing system based on UNIX pipes. J Biomol NMR. 1995; 6:277–93. 10.1007/BF00197809. 8520220

[42] Kobayashi N, Iwahara J, Koshiba S et al. KUJIRA, a package of integrated modules for systematic and interactive analysis of NMR data directed to high-throughput NMR structure studies. J Biomol NMR. 2007; 39:31–52. 10.1007/s10858-007-9175-5. 17636449

[43] Ahlner A, Carlsson M, Jonsson BH et al. PINT: a software for integration of peak volumes and extraction of relaxation rates. J Biomol NMR. 2013; 56:191–202. 10.1007/s10858-013-9737-7. 23657843

[44] Shimizu N, Mori T, Nagatani Y et al. BL-10C, the small-angle X-ray scattering beamline at the photon factory. AIP Conf Proc. 2019; 2054:060041 10.1063/1.5084672.

[45] Takagi H, Igarashi N, Nagatani Y et al. New high-brilliance small angle X-ray scattering beamline, BL-15A2 at the photon factory. AIP Conf Proc. 2019; 2054:060038 10.1063/1.5084669.

[46] Shimizu N, Yatabe K, Nagatani Y et al. Software development for analysis of small-angle X-ray scattering data. AIP Conf Proc. 2016; 1741:050017 10.1063/1.4952937.

[47] Yonezawa K, Takahashi M, Yatabe K et al. MOLASS: software for automatic processing of matrix data obtained from small-angle X-ray scattering and UV-visible spectroscopy combined with size-exclusion chromatography. BIOPHYSICS. 2023; 20:e200001 10.2142/biophysico.bppb-v20.0001. PMC10203098 37229310

[48] Petoukhov MV, Konarev PV, Kikhney AG et al. ATSAS 2.1—towards automated and web-supported small-angle scattering data analysis. J Appl Crystallogr. 2007; 40:s223–8. 10.1107/S0021889807002853.

[49] Manalastas-Cantos K, Konarev PV, Hajizadeh NR et al. ATSAS 3.0: expanded functionality and new tools for small-angle scattering data analysis. J Appl Crystallogr. 2021; 54:343–55. 10.1107/S1600576720013412. 33833657 PMC7941305

[50] Tria G, Mertens HD, Kachala M et al. Advanced ensemble modelling of flexible macromolecules using X-ray solution scattering. IUCrJ. 2015; 2:207–17. 10.1107/S205225251500202X. PMC4392415 25866658

[51] Kaustov L, Ouyang H, Amaya M et al. Recognition and specificity determinants of the human cbx chromodomains. J Biol Chem. 2011; 286:521–9. 10.1074/jbc.M110.191411. 21047797 PMC3013012

[52] Kang J, Chaudhary J, Dong H et al. Mitotic centromeric targeting of HP1 and its binding to Sgo1 are dispensable for sister-chromatid cohesion in human cells. MBoC. 2011; 22:1181–90. 10.1091/mbc.e11-01-0009. 21346195 PMC3078076

[53] Kikhney AG, Borges CR, Molodenskiy DS et al. SASBDB: towards an automatically curated and validated repository for biological scattering data. Protein Sci. 2020; 29:66–75. 10.1002/pro.3731. 31576635 PMC6933840

[54] Jumper J, Evans R, Pritzel A et al. Highly accurate protein structure prediction with AlphaFold. Nature. 2021; 596:583–9. 10.1038/s41586-021-03819-2. 34265844 PMC8371605

[55] Sali A, Blundell TL Comparative protein modelling by satisfaction of spatial restraints. J Mol Biol. 1993; 234:779–815. 10.1006/jmbi.1993.1626. 8254673

[56] Marrink SJ, Risselada HJ, Yefimov S et al. The MARTINI force field: coarse grained model for biomolecular simulations. J Phys Chem B. 2007; 111:7812–24. 10.1021/jp071097f. 17569554

[57] Monticelli L, Kandasamy SK, Periole X et al. The MARTINI Coarse-grained force field: extension to proteins. J Chem Theory Comput. 2008; 4:819–34. 10.1021/ct700324x. 26621095

[58] De Jong DH, Singh G, Bennett WFD et al. Improved parameters for the martini coarse-grained protein force field. J Chem Theory Comput. 2013; 9:687–97. 10.1021/ct300646g. 26589065

[59] Martinez L, Andrade R, Birgin EG et al. PACKMOL: a package for building initial configurations for molecular dynamics simulations. J Comput Chem. 2009; 30:2157–64. 10.1002/jcc.21224. 19229944

[60] Bussi G, Donadio D, Parrinello M Canonical sampling through velocity rescaling. J Chem Phys. 2007; 126:014101 10.1063/1.2408420. 17212484

[61] Parrinello M, Rahman A Polymorphic transitions in single crystals: a new molecular dynamics method. J Appl Phys. 1981; 52:7182–90. 10.1063/1.328693.

[62] Tironi IG, Sperb R, Smith PE et al. A generalized reaction field method for molecular dynamics simulations. J Chem Phys. 1995; 102:5451–9. 10.1063/1.469273.

[63] Hess B, Bekker H, Berendsen H et al. LINCS: a linear constraint solver for molecular simulations. J Comput Chem. 1997; 18:1463–72. 10.1002/(SICI)1096-987X(199709)18:12<1463::AID-JCC4>3.0.CO;2-H.

[64] Hess B P-LINCS: a parallel linear constraint solver for molecular simulation. J Chem Theory Comput. 2008; 4:116–22. 10.1021/ct700200b. 26619985

[65] Abraham MJ, Murtola T, Schulz R et al. GROMACS: high performance molecular simulations through multi-level parallelism from laptops to supercomputers. SoftwareX. 2015; 1-2:19–25. 10.1016/j.softx.2015.06.001.

[66] Bottaro S, Bengtsen T, Lindorff-Larsen K Integrating molecular simulation and experimental data: a bayesian/maximum entropy reweighting approach. Methods Mol Biol. 2020; 2112:219–40. 10.1007/978-1-0716-0270-6_15. 32006288

[67] Wassenaar TA, Pluhackova K, Bockmann RA et al. Going backward: a flexible geometric approach to reverse transformation from coarse grained to atomistic models. J Chem Theory Comput. 2014; 10:676–90. 10.1021/ct400617g. 26580045

[68] Schneidman-Duhovny D, Hammel M, Tainer JA et al. FoXS, FoXSDock and MultiFoXS: single-state and multi-state structural modeling of proteins and their complexes based on SAXS profiles. Nucleic Acids Res. 2016; 44:W424–9. 10.1093/nar/gkw389. 27151198 PMC4987932

[69] Sadaie M, Kawaguchi R, Ohtani Y et al. Balance between distinct HP1 family proteins controls heterochromatin assembly in fission yeast. Mol Cell Biol. 2008; 28:6973–88. 10.1128/MCB.00791-08. 18809570 PMC2593388

[70] Nishibuchi G, Machida S, Nakagawa R et al. Mitotic phosphorylation of HP1alpha regulates its cell cycle-dependent chromatin binding. J Biochem. 2019; 165:433–46. 10.1093/jb/mvy117. 30590679

[71] Watanabe S, Mishima Y, Shimizu M et al. Interactions of HP1 bound to H3K9me3 dinucleosome by molecular simulations and biochemical assays. Biophys J. 2018; 114:2336–51. 10.1016/j.bpj.2018.03.025. 29685391 PMC6129468

[72] Mishima Y, Watanabe M, Kawakami T et al. Hinge and chromoshadow of HP1alpha participate in recognition of K9 methylated histone H3 in nucleosomes. J Mol Biol. 2013; 425:54–70. 10.1016/j.jmb.2012.10.018. 23142645

[73] Hayakawa T, Haraguchi T, Masumoto H et al. Cell cycle behavior of human HP1 subtypes: distinct molecular domains of HP1 are required for their centromeric localization during interphase and metaphase. J Cell Sci. 2003; 116:3327–38. 10.1242/jcs.00635. 12840071

[74] Matsui S, Nozawa RS RNA impacts formation of biomolecular condensates in the nucleus. Biomed Res. 2021; 42:153–60. 10.2220/biomedres.42.153. 34380923

