# Supplementary material for: A dynamic structural unit of phase-separated heterochromatin protein 1α as revealed by integrative structural analyses
Source: Nucleic Acids Res. 2025 Mar 24;53(6):gkaf154. doi: 10.1093/nar/gkaf154 (PMC11930357; doi:10.1093/nar/gkaf154)
Supplement: gkaf154_Supplemental_Files [file gkaf154_supplemental_files.zip › 250128Supplementary Figures1.docx]

**SUUPLEMENTARY FIGUREs**

**Supplementary Figure 1. NMR spectra of HP1α and p HP1α.**

(**A**, **B**) Superposition of ^1^H-^15^N HSQC spectra of HP1α (blue) and pHP1α (red) at 500 mM NaCl (**A**) and 50 mM NaCl (**B**). Both HP1α and pHP1α were measured at a concentration of 150 μM dimer. Inserts show expanded ^1^H-^15^N HSQC spectra. (**C**) Chemical shift differences (⊿δ) between HP1α and pHP1α at 500 and 50 mM NaCl. Purple line shows 500 mM NaCl; Brawn line shows 50 mM NaCl; green and magenta broken lines show average Δδ and average plus SD Δδ, respectively. (**D**) Mapping of residues showing significant Δδ in panel **C** on the HP1α model structure generated using Modeller 9. Residues whose Δδ value was larger than the average and the average plus SD are colored green and magenta, respectively.

**Supplementary Figure 2. Backbone {^1^H}-^15^N heteronuclear NOE values of HP1α (blue) and pHP1α (red) at 50 mM NaCl.**

**
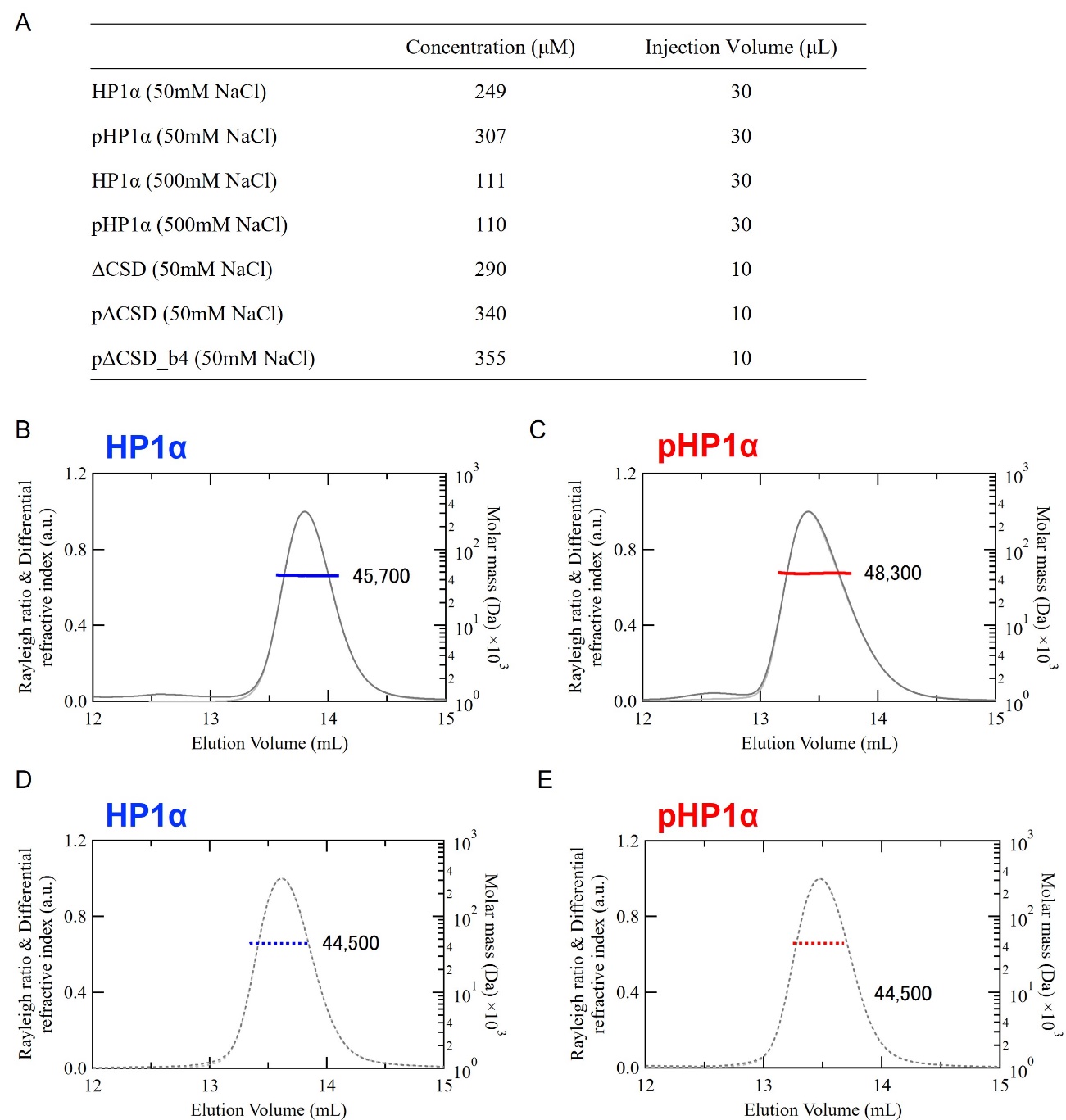
**

**Supplementary Figure 3. SEC-MALS conditions and results of HP1α and pHP1α.**

(**A**) Concentration and volume of samples injected into the HPLC system. (**B–E**) SEC-MALS results for HP1α and pHP1α. The gray and light gray lines indicate the Rayleigh ratio and the differential refractive index, respectively, and the blue and red lines correspond to the molar mass of HP1α and pHP1α, respectively, with the variation in salt concentration denoted by the solid and dotted lines. (**B**) HP1α (50 mM NaCl); (**C**) pHP1α (50 mM NaCl); (**D**) HP1α (500 mM NaCl); (**E**) pHP1α (500 mM NaCl).

**
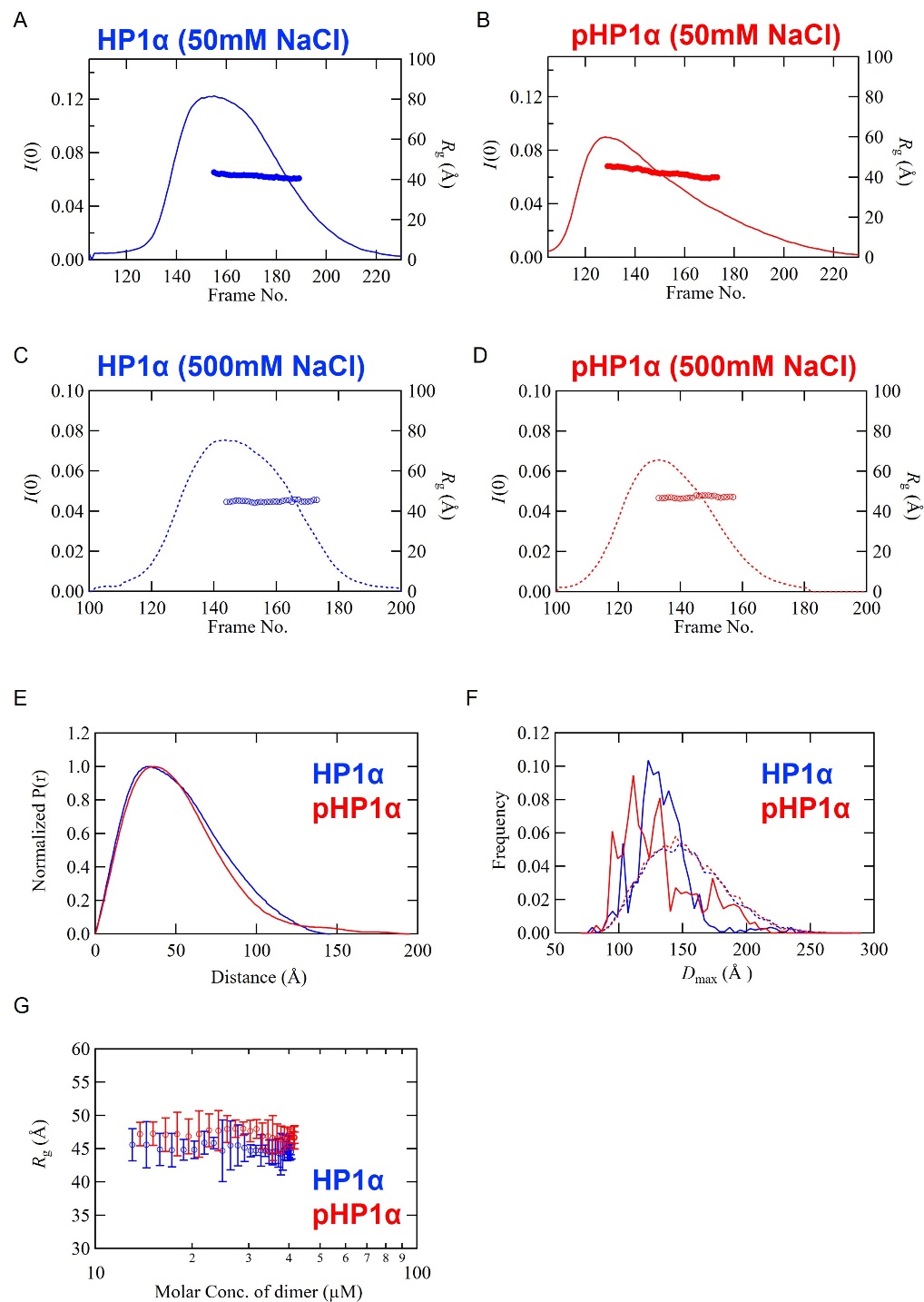
**

**Supplementary Figure 4. SEC-SAXS measurements of HP1α and pHP1α at 50 and 500 mM NaCl.**

(**A–D**) Distribution plots of *I*(0) (solid and dotted lines) and *R*_g_ (open and closed circles) obtained from Guinier analysis. The range shown in the *R*_g_ distribution indicates the data range used for analysis. (**A**) HP1α (50 mM NaCl); (**B**) pHP1α (50 mM NaCl); (**C**) HP1α (500 mM NaCl); (**D**) pHP1α (500 mM NaCl). (**E**) *P*(r) functions of HP1α (blue) and pHP1α (red) calculated from the experimental SAXS profiles shown in Fig. 2B. (**F**) *D*_max_ distributions derived from EOM analysis of HP1α (blue) and pHP1α (red). Solid and dashed lines show the distributions after EOM calculation and the initial distributions before the calculation, respectively. (**G**) *R*_g_ values (**C**, **D**) plotted against molar dimer concentration for HP1α (blue) and pHP1α (red) at 500 mM NaCl.

**Supplementary Figure 5. Comparison of HP1α and pHP1α by GCMD-SAXS.**

(**A**, **B**) Fit of the SAXS profiles calculated with the reweighted ensembles to the experimental data for HP1α (**A**) and pHP1α (**B**). (**C**) Distributions of *D*_max_ calculated from the reweighted ensembles.

**Supplementary Figure 6. Representative structures of the top 10 clusters, which account for 28.8 % of all structures, from the weighted ensembles of HP1α.**

**Supplementary Figure 7. Representative structures of the top 10 clusters, which account for 45.7 % of all structures, from the weighted ensembles of pHP1α.**

**
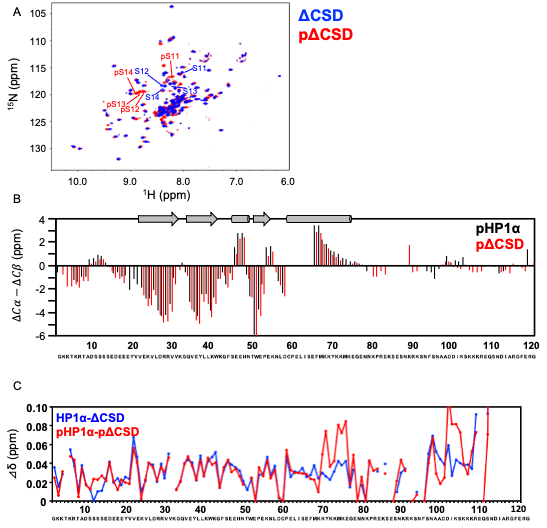
Supplementary Figure 8. Comparison between HP1α and ΔCSD by NMR.**

1. (**A**) Superposition of ^1^H-^15^N HSQC spectra of ΔCSD (blue) and pΔCSD (red) at 50 mM NaCl.

(**B**) Secondary structure from chemical shift index data. Shown are the chemical shift indices of pHP1α (black) and pΔCSD (red). The chemical shift index of each residue at the i-th position was calculated as ΔCα–ΔCβ= [{Cα (i − 1) + Cα (i) + Cα (i + 1) - {Cβ (i − 1) + Cβ (i) + Cβ (i + 1)}]/3. (**C**) Chemical shift differences (⊿δ) between HP1α and ΔCSD (blue) and between pHP1α and pΔCSD (red) at 50 mM NaCl.

**
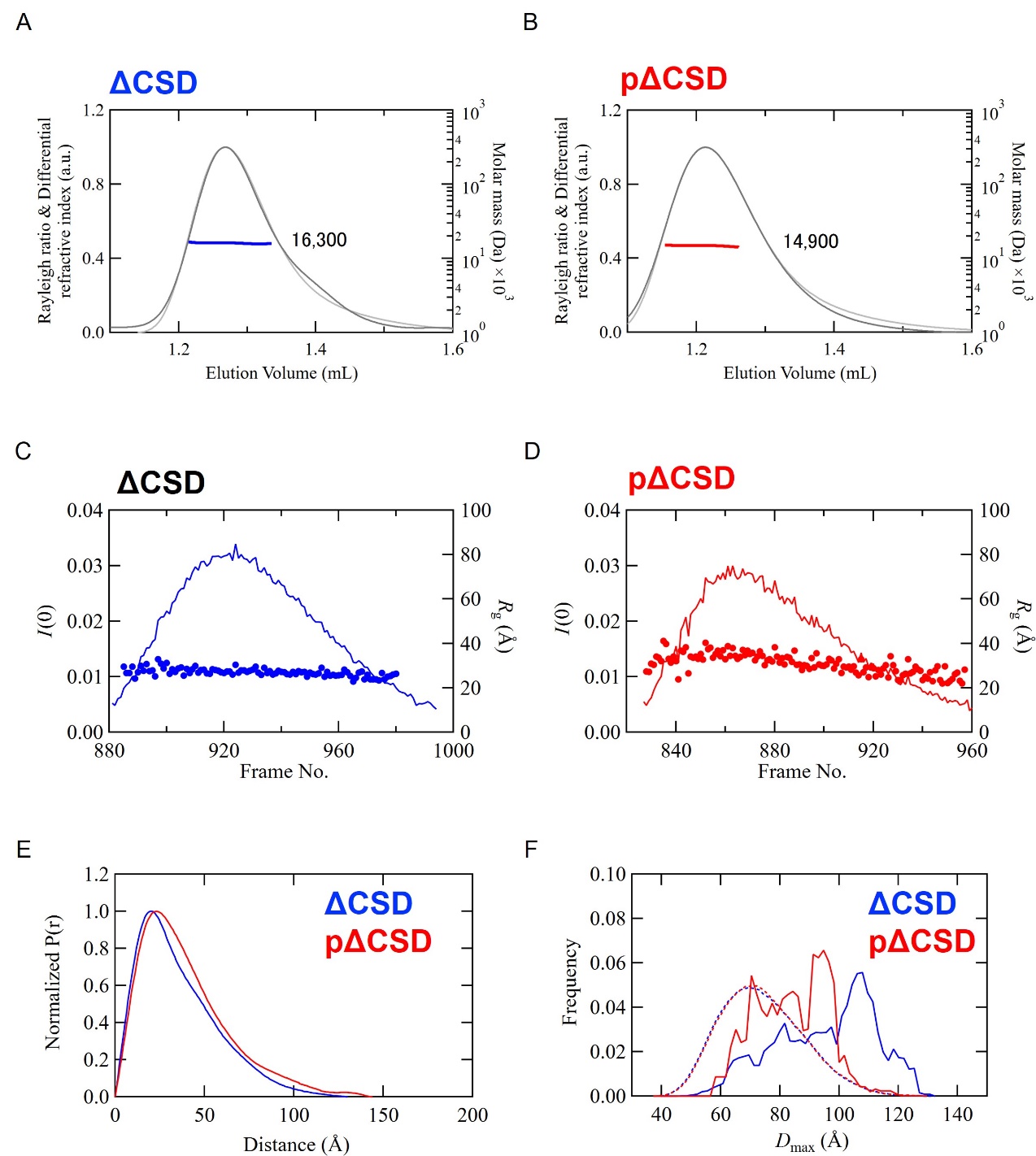
**

**Supplementary Figure 9. SEC-MALS/SAXS and EOM analysis of ΔCSD mutants.**

(**A**,**B**) SEC-MALS results for ΔCSD (**A**) and pΔCSD (**B**) at 50 mM NaCl. Gray, light gray, and blue/red lines display the Rayleigh ratio, differential refractive index, and molar mass, respectively. (**C**, **D**) Distribution plots of *I*(0) (blue or red lines) and *R*_g_ (blue or red closed circles) resulting from Guinier analysis in SEC-SAXS measurements performed at 50 NaCl. The range shown in the *R*_g_ distribution indicates the data range used for the analysis. (**C**) ΔCSD; (**D**) pΔCSD. (**E**) *P*(r) functions of ΔCSD (blue) and pΔCSD (red) calculated based on the experimental SAXS profiles shown in Fig. 4B. (**F**) *D*_max_ distributions derived from EOM analysis of ΔCSD (blue) and pΔCSD (red). Solid and dashed lines show the distributions after EOM calculation and the initial distributions before the calculation, respectively.

**Supplementary Figure 10. Comparison of ΔCSD and pΔCSD by GCMD-SAXS.**

(**A–D**) Fit of the calculated SAXS profiles to the experimental data. Profiles were calculated with the reweighted ensembles from simulations of the single-molecule system of ΔCSD (**A**), two-molecule system of ΔCSD (**B**), single-molecule system of pΔCSD (**C**), and two-molecule system of pΔCSD (**D**).

**Supplementary Figure 11.** Representative structures of the top 10 clusters, which account for 61 % of all structures, from the weighted ensemble from the simulation of two-molecule system of pΔCSD.

**Supplementary Figure 12. LLPS of pHP1α and pΔCSD.**

(**A**) pHP1α solution appears as two separate phases in the NMR tube at 298K, 50 mM NaCl, 20 mM sodium phosphate buffer (pH 7.0), 1 mM DTT. (**B**) Micrograph of pHP1α. Left. 500 μM Scale bar, 100 μm. Right. 150 μM Scale bar, 10 μm. (**C**) Micrograph of pΔCSD. Scale bar, 10 μm. (**D**) Photographs captured with the CCD camera used to observe the sample cell in the SAXS experiment. Images depict pΔCSD and pΔCSD_b4 in 50 mM NaCl, corresponding to the conditions of the original solutions shown in Supplementary Fig. 3A.**S****upplementary Figure 13. Comparison of pΔCSD between dilute and condensed solutions.**

(**A**) Superposition of the ^1^H-^15^N HSQC spectra of pΔCSD in dilute (120 μM, red) and condensed (400 μM, black) solutions. (**B**) Intensity of each signal of pΔCSD in dilute solution (120 μM, red) multiplied by a factor 3.33 (400/120) to allow comparison of the corresponding signal intensity of pΔCSD in condensed solution (400 μM, black).

**Supplementary Figure 14.** Secondary structure determined from chemical shift index data. Shown are the chemical shift indices of pΔCSD (red) and pΔCSD _b4 (black).


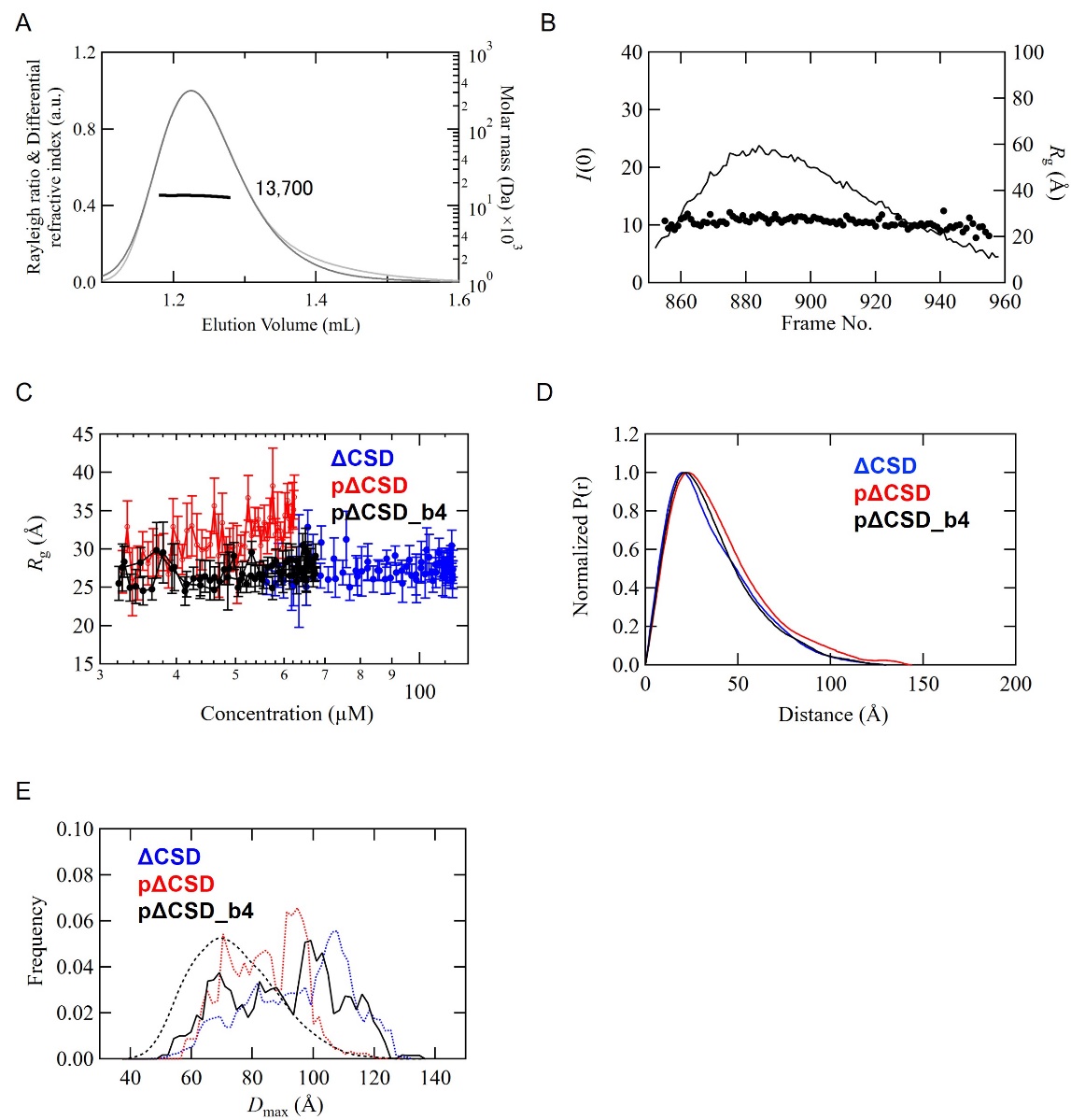


**Supplementary Figure 15. SEC-MALS analysis of CSD mutants.**

(**A**) SEC-MALS results for the ΔCSD_b4 mutant measured at 50 mM NaCl. Gray, light gray, and black lines display the Rayleigh ratio, differential refractive index, and molar mass, respectively. (**B**) Distribution plots of *I*(0) (black line) and *R*_g_ (black closed circle) resulting from Guinier analysis in SEC-SAXS measurements performed at 50 mM NaCl. The range shown in the *R*_g_ distribution indicates the data range used in the analysis. (**C**) Plot of the *R*_g_ values in panel **B** against molar concentration (black circles). For comparison, the ΔCSD (blue circles) and pΔCSD (red circles) values from Fig. 4B are also plotted. (**D**) *P*(r) functions of ΔCSD (blue), pΔCSD (red), and ΔCSD_b4 (black) calculated based on the experimental SAXS profiles shown in Fig. 4C and 5D; for comparison, the functions of ΔCSD and pΔCSD are reproduced from Supplementary Fig. 9E. (**E**) *D*_max_ distribution derived from EOM analysis. Black solid and dashed lines for ΔCSD_b4 show the distribution after EOM calculation and the initial distribution before the calculation, respectively. Blue and red dotted lines represent the results of ΔCSD and pΔCSD, respectively; for comparison, these are reproduced from Supplementary Fig. 9F.

**Supplementary Figure 16.** pLDDT scores for model structures of (**A**) CD (residues 16–80) and (**B**) CSD (residues 111–180) predicted by Alphafold2.

**Supplementary Figure 17.** Plot of χ^2^ scores vs $\varphi_{\mathrm{eff}}$ for *θ* = (1, 5, 10, 20, 50, 100, 500, 1000) using snapshots of simulations of (**A**) HP1α, (**B**) pHP1α, (**C**) the single-molecule system of ΔCSD, (**D**) the two-molecule system of ΔCSD, (**E**) the single-molecule system of pΔCSD, and (**F**) the two-molecule system of pΔCSD.

**Supplementary Figure 18. Comparison of NMR signals of pHP1α in the dilute and the condensed solutions.**

Shown are normalized signal intensities of pHP1α in the dilute (150 μM, red) and condensed (500 μM, black) solution. Signal intensities were normalized by the respective Ser191 signal intensity. Red and black crosses indicate unassigned residues of pHP1α.
