# Supplementary material for: A dynamic structural unit of phase-separated heterochromatin protein 1α as revealed by integrative structural analyses
Source: Nucleic Acids Res. 2025 Mar 24;53(6):gkaf154. doi: 10.1093/nar/gkaf154 (PMC11930357; doi:10.1093/nar/gkaf154)
Supplement: gkaf154_Supplemental_Files [file gkaf154_supplemental_files.zip › 250128Supplementary Tables.docx]

**Supplementary Table S1.**

**Supplementary Table S2. List of primers used in this study**

| Name Sequence Experiment |
| --- |

ura4-RT-Fw1 5’-GGC CTC AAA GAA GTT GGT TTA CC-3’ RT-qPCR

ura4-RT-Rv1 5’-GAA GAC ATT TCA GCC AAA AGC A-3’ RT-qPCR

act1_RT_Fw 5’-CGT GCC CCT GAA GCT CTT T-3’ RT-qPCR

act1_RT_Rv 5’-CTC ATG AAT ACC GGC GTT TTC-3’ RT-qPCR

|  |
| --- |
