# Supplementary material for: A dynamic structural unit of phase-separated heterochromatin protein 1α as revealed by integrative structural analyses
Source: Nucleic Acids Res. 2025 Mar 24;53(6):gkaf154. doi: 10.1093/nar/gkaf154 (PMC11930357; doi:10.1093/nar/gkaf154)
Supplement: gkaf154_Supplemental_Files [file gkaf154_supplemental_files.zip › 250128Suulementary Data.docx]

**SUPPLEMENTARY DATA**

**Assessment of the structural stability of the domain structures in coarse-grained (CG) molecular dynamics (MD) simulations**

The structures of residues 19–74 of chromodomain (CD) and residues 113–173 of chromoshadow domain (CSD) were predicted with a high confidence score (pLDDT > 70) by AlphaFold2 (1) (Supplementary Figure 16). In the CGMD simulations, therefore, the structures of these regions were maintained by elastic networks. We assessed the stabilities of the model structures of CD (residues 16‒80) and CSD dimer (residues 111‒180) in the CGMD simulations with the MARTINI 2.2 force field (2,3) and compared them with the results of all-atom (AA) MD simulations. Systems for CGMD simulations were constructed by solvating a protein in a box of ~8,900 CG water molecules and adding Na^+^ and Cl^−^ beads at a concentration of 50 mM. The protein structures were coarse-grained using the martinize.py script (4). Backbone bead pairs at a distance of 5–11 Å within CD and the CSD dimer were connected by harmonic bonds with force constants of 250 and 150 kJ mol^−1^ nm^−2^, respectively. Energy minimization, 200-ns equilibration, and a 2-µs production run were performed for each model. Other settings for CGMD simulations were the same as those described in the “Methods” section. For AAMD simulations, the model structures of CD and CSD were solvated in boxes of ~19,000 and ~27,000 water molecules, respectively. Na^+^ and Cl^−^ ions were added to each system at a concentration of 50 mM. After energy minimizations, the systems were heated to 300 K for 200 ps. During this simulation, harmonic position restraints with a force constant of 1000 kcal mol^−1^ nm^−2^ were imposed on the Cα atoms of the protein. Next, the systems were equilibrated in the constant-*NPT* ensemble at 300 K and 1.0 bar, for 2.0 ns. The force constant of the restraints was gradually reduced from 1000 to 0 kcal mol^−1^ nm^−2^ during the equilibration. Subsequently, 200-ns production runs were performed in the constant-*NPT* ensembles. The Amber ff14SB (5) force field was used for the proteins and the TIP3P model was used for the water molecules (6). Temperature and pressure were controlled by using a velocity-rescaling thermostat (7) and a Parrinello–Rahman barostat (8), respectively. Electrostatic interactions were calculated by the particle mesh Ewald method (9,10) using a real space cutoff of 0.8 nm. Van der Waals interactions were calculated by a modified Lennard-Jones potential using a cutoff distance of 0.8 nm. Bond lengths related to the hydrogen atoms were constrained by the linear constraint solver (LINCS) algorithm (11,12) to allow the use of a 2-fs time step. The distributions of root mean-square deviations (RMSDs) and root mean-square fluctuations (RMSFs) were calculated for the backbone beads of the CGMD snapshots and for the Cα atoms of the AAMD snapshots (Supplementary Data Figure 1). The results of RMSD showed that the structural deviations from the initial structure in the CGMD simulations are comparable to those in the AAMD simulations. The results of RMSF suggested that the structural fluctuations in the CGMD simulations are also comparable to those in the AAMD simulations.

**Parameterization of phosphorylated serine**

Phosphorylated serine was mapped to a single backbone bead and a single sidechain bead. AQa particle with a charge of −2 was used for the sidechain bead to represent the chemical properties of the phosphate. The type of backbone bead was set the same as the serine residue. The length and force constant for the bond between the backbone and sidechain beads were set to 0.427 nm and 6,000 kJ mol^−1^ nm^−2^, respectively, based on a comparison of CGMD and AAMD simulations for phosphorylated serine models in water. The AAMD simulation was performed using an Amber ff14SB force field and the TIP3P water model. The parameter for phosphorylated serine was taken from the literature (13). An energy minimization, a 0.1-ps heating, a 0.8-ns equilibration, and a 200 ns production run were performed with the same settings described in the previous section. The distributions of the bond length of the CG model of phosphorylated serine agreed with those calculated from the CG-mapped trajectory of the AAMD simulation (Supplementary Data Figure 2).

**Tuning protein-solvent interactions of the MARTINI force field**

The MARTINI 2.2 force field parameters were modified because they tend to overestimate protein–protein interactions. Several previous studies have attempted to alleviate the overestimation by scaling protein–protein interactions or protein–water interactions (14-16). With reference to a previous study (16), we increased the strength of interactions between protein and solvents by multiplying the *ε* values of the Lennard–Jones potential between protein beads and water/ion beads by a factor of *λ*. We performed a series of simulations in which we changed *λ* from 1.04 to 1.10 to examine the *λ*-dependencies of the radius of gyrations (*R*_g_) and fitting scores (*χ*^2^) calculated from the reweighted ensembles by BME with *θ* = 20 for the HP1α and pHP1α dimers (Supplementary Data Table S1). For HP1α, the averaged *R*_g_ was closest at *λ* = 1.075, with a deviation of −1.4 Å from the experimental value (39.8 Å) and the *χ*^2^ value were also small enough to be comparable to the result of EOM, *χ*^2^ = 1.396. In addition, the effective fraction ($\varphi_{\mathrm{eff}}$) was large, which indicates that a large fraction of the snapshots effectively contributed to the reweighted ensemble obtained by BME and suggests that the structural ensemble generated by the CGMD simulation under this condition (*λ* = 1.075) agreed well with the actual ensemble analyzed by the experiment (16). For pHP1α, the deviation from the experimental value (38.1 Å) was +1.6 Å at *λ* = 1.075, which was the second closest to the experimental value after *λ* = 1.08 (−0.7 Å). The small *χ*^2^ scores (comparable to the result of EOM, *χ*^2^ = 1.216) and large $\varphi_{\mathrm{eff}}$ values were also obtained for both *λ* = 1.075 and *λ* = 1.08. Overall, when *λ* = 1.075, structural ensembles that agree well with the experimental SAXS data can be obtained for both of HP1α and pHP1α. We therefore used *λ* = 1.075 for all the CGMD simulations.

**Supplementary Data Figure 1.** Comparison of the distributions of (**A, C**) RMSD and (**B, D**) RMSF between AAMD (blue) and CGMD (orange) simulations for (**A, B**) residues 19–74 of CD and (**C, D**) residues 113–173 of the CSD dimer. The RMSD and the RMSF values were calculated for the backbone beads of the CGMD snapshots and for the Cα atoms of the AAMD snapshots.

**Supplementary Data Figure 2.** Comparison of the probability distributions of the backbone–sidechain distance of phosphorylated serine between AAMD (blue) and CGMD (orange) simulations. For AAMD, the distance was measured between the center of mass of the backbone atoms and that of the side chain atoms. For CGMD, the distance was measured between the backbone and the sidechain CG beads.

**Supplementary Data Table S1. Averaged value of *R*_g_, fitness score (*χ*^2^), and effective fraction (*φ*_eff_) at different values of λ**

|  | **HP1α** |  |  | **pHP1α** |  |  |
| --- | --- | --- | --- | --- | --- | --- |
|  | *R*_g_ (Å) | *χ*^2^ | *φ*_eff_ | *R*_g_ (Å) | *χ*^2^ | *φ*_eff_ |
| 1.04 | 28.9 | 1.430 | 0.01 | 27.1 | 1.227 | 0.03 |
| 1.06 | 33.5 | 1.402 | 0.68 | 32.4 | 1.210 | 0.42 |
| 1.07 | 37.7 | 1.387 | 0.63 | 36.5 | 1.215 | 0.73 |
| 1.075 | 40.5 | 1.388 | 0.78 | 36.8 | 1.216 | 0.83 |
| 1.08 | 44.9 | 1.378 | 0.47 | 38.8 | 1.207 | 0.84 |
| 1.09 | 47.1 | 1.411 | 0.27 | 46.3 | 1.235 | 0.39 |
| 1.10 | 50.4 | 1.444 | 0.06 | 49.0 | 1.253 | 0.17 |
| Experimental | 39.8 |  |  | 38.1 |  |  |

**References**

1. Jumper, J., Evans, R., Pritzel, A., Green, T., Figurnov, M., Ronneberger, O., Tunyasuvunakool, K., Bates, R., Žídek, A., Potapenko, A., et al. (2021) Highly accurate protein structure prediction with AlphaFold. *Nature*, **596**, 583–589.
2. Marrink, S.J., Risselada, H.J., Yefimov, S., Tieleman, D.P. and de Vries, A.H. (2007) The MARTINI force field: coarse grained model for biomolecular simulations. *J Phys Chem B*, **111**, 7812-7824.
3. Monticelli, L., Kandasamy, S.K., Periole, X., Larson, R.G., Tieleman, D.P. and Marrink, S.J. (2008) The MARTINI Coarse-Grained Force Field: Extension to Proteins. *J Chem Theory Comput*, **4**, 819-834.
4. De Jong, D.H., Singh, G., Bennett, W.F.D., Arnarez,C., Wassenaar, T.A., Schäfer, L. V., Periole, X., Tieleman, D.P. and Marrink, S.J. (2013) Improved parameters for the martini coarse-grained protein force field. *J Chem Theory Comput*, **9**, 687–697.
5. Maier, J.A., Martinez, C., Kasavajhala, K., Wickstrom, L., Hauser, K.E. and Simmerling, C. (2015) ff14SB: Improving the Accuracy of Protein Side Chain and Backbone Parameters from ff99SB. *J Chem Theory Comput*, **11**, 3696-3713.
6. Jorgensen, W.L., Chandrasekhar J, Madura, J.D., Impey, R.W. and Klein, M.L. (1983) Comparison of simple potential functions for simulating liquid water. *Journal of Chemical Physics,* **79**, 926-935.
7. Bussi, G., Donadio, D. and Parrinello, M. (2007) Canonical sampling through velocity rescaling. *J Chem Phys*, **126**, 014101.
8. Parrinello, M. and Rahman, A. (1981) Polymorphic Transitions in Single Crystals: A New Molecular Dynamics Method. *J Appl Phys,* **52**, 7182–7190. doi:10.1063/1.328693
9. Essmann, U., Perera, L., Berkowitz, M.L., Darden, T., Lee, H. and Pedersen, L.G. (1995) A smooth particle mesh Ewald method. *Journal of Chemical Physics,* **103**, 8577-8593.
10. Darden, T., York, D. and Pedersen, L. (1993) An N⋅log(N) method for Ewald sums in large systems. *Journal of Chemical Physics,* **98**, 10089-10092.
11. Hess, B., Bekker, H., Berendsen, H. J. C. and Fraaije, J. G. E. M. (1997) LINCS: A linear constraint solver for molecular simulations. *J Comput Chem,* ***18***, 1463-1472.
12. Hess, B. (2007) P-LINCS: A Parallel Linear Constraint Solver for Molecular Simulation. *J Chem Theory Comput,* ***4***, 116-122.
13. Homeyer, N., Horn, A.H., Lanig, H. and Sticht, H. (2006) AMBER force-field parameters for phosphorylated amino acids in different protonation states: phosphoserine, phosphothreonine, phosphotyrosine, and phosphohistidine. *J Mol Model*, **12**, 281-289.
14. Stark, A.C., Andrews, C.T. and Elcock, A.H. (2013) Toward optimized potential functions for protein-protein interactions in aqueous solutions: osmotic second virial coefficient calculations using the MARTINI coarse-grained force field. *J Chem Theory Comput*, **9**, 4176-4185
15. Javanainen, M., Martinez-Seara, H. and Vattulainen, I. (2017) Excessive aggregation of membrane proteins in the Martini model. *PLoS One*, **12**, e0187936.
16. Larsen, A.H., Wang, Y., Bottaro, S., Grudinin, S., Arleth, L. and Lindorff-Larsen, K. (2020) Combining molecular dynamics simulations with small-angle X-ray and neutron scattering data to study multi-domain proteins in solution. *PLoS Comput Biol*, **16**, e1007870.
